# The protective role of nutritional antioxidants against oxidative stress in thyroid disorders

**DOI:** 10.3389/fendo.2022.1092837

**Published:** 2023-01-04

**Authors:** Mirjana T. Macvanin, Zoran Gluvic, Sonja Zafirovic, Xin Gao, Magbubah Essack, Esma R. Isenovic

**Affiliations:** ^1^ Department of Radiobiology and Molecular Genetics, VINČA Institute of Nuclear Sciences - National Institute of the Republic of Serbia, University of Belgrade, Belgrade, Serbia; ^2^ Clinic for Internal Medicine, Department of Endocrinology and Diabetes, Zemun Clinical Hospital, School of Medicine, University of Belgrade, Belgrade, Serbia; ^3^ Computational Bioscience Research Center (CBRC), King Abdullah University of Science and Technology (KAUST), Thuwal, Saudi Arabia; ^4^ Computer Science Program, Computer, Electrical and Mathematical Sciences and Engineering Division (CEMSE), King Abdullah University of Science and Technology (KAUST), Thuwal, Saudi Arabia

**Keywords:** oxidative stress, reactive oxygen species, thyroid disease, nutritional antioxidants, thyroid-gut axis, gut microbiome, antioxidant probiotic

## Abstract

An imbalance between pro-oxidative and antioxidative cellular mechanisms is oxidative stress (OxS) which may be systemic or organ-specific. Although OxS is a consequence of normal body and organ physiology, severely impaired oxidative homeostasis results in DNA hydroxylation, protein denaturation, lipid peroxidation, and apoptosis, ultimately compromising cells’ function and viability. The thyroid gland is an organ that exhibits both oxidative and antioxidative processes. In terms of OxS severity, the thyroid gland’s response could be physiological (i.e. hormone production and secretion) or pathological (i.e. development of diseases, such as goitre, thyroid cancer, or thyroiditis). Protective nutritional antioxidants may benefit defensive antioxidative systems in resolving pro-oxidative dominance and redox imbalance, preventing or delaying chronic thyroid diseases. This review provides information on nutritional antioxidants and their protective roles against impaired redox homeostasis in various thyroid pathologies. We also review novel findings related to the connection between the thyroid gland and gut microbiome and analyze the effects of probiotics with antioxidant properties on thyroid diseases.

## 1 Introduction

Cellular redox homeostasis depends on a dynamic equilibrium between prooxidant production and its elimination. Reactive oxygen species (ROS), together with reactive nitrogen species (RNS), represent the most important prooxidants whose excessive accumulation leads to oxidative stress (OxS) and molecular damage (1, 2). ROS are molecules with an oxygen atom, and unpaired electrons are primarily generated as by-products of ATP synthesis in mitochondrial respiratory chains (1) or during inflammation (3, 4). The concentration of ROS determines their physiological role (5). When present at low concentrations, ROS are involved in signaling processes essential for normal cellular functions (6, 7), whereas high ROS concentration leads to DNA, lipid, and protein damage and apoptosis (8).

ROS are crucial in thyroid function because they are essential in the initial stages of thyroid hormone synthesis during iodide oxidation (9). Also, the process whereby thyroid peroxidase (TPO) catalyzes thyroxine (T4) and triiodothyronine (T3) during its synthesis in thyroid follicles involves ROS (10). In addition, thyroid hormones affect the mitochondrial activity and modulate ROS production (10). The dependence of normal thyroid function on ROS implies that the thyroid is continuously exposed to ROS and, thus, particularly sensitive to oxidative damage (10). Therefore, to protect the integrity of the thyroid, it is mandatory that the thyroid antioxidant defence system effectively regulates and balances ROS production and elimination (11, 12).

Aerobic organisms have evolved multiple antioxidant and repair systems for protection against OxS. Enzymes that decompose ROS, such as catalase (CAT), superoxide dismutase (SOD), glutathione peroxidase (GPx), and glutathione reductase (GR) provide the primary antioxidant defence (5, 7, 8, 13). In contrast, ROS-induced damage repair systems eliminate damaged cells through autophagy and apoptosis processes (14, 15). However, the capacity of intrinsic antioxidant systems is not always sufficient to prevent damage caused by excessive accumulation of ROS. Thus, non-enzymatic mechanisms based on the action of molecules with antioxidant properties such as glutathione (GSH), thioredoxin, coenzyme Q10, and exogenous antioxidants, including various polyphenolic compounds, ascorbic acid, tocopherol retinol, and β-carotene, that may also support antioxidant systems are essential. The use of nutritional antioxidants as supplementary substances that delay and/or prevent the oxidation of cellular components has shown the potential to protect human organs, including the thyroid gland, against oxidative damage by reinforcing the body’s antioxidant defence and increasing total antioxidant capacity (5, 16, 17).

Recently, a search for natural nutritional antioxidants from biological resources has gained substantial attention. Of particular interest are probiotics which represent live non-pathogenic microorganisms that can restore microbial balance in the gastrointestinal tract upon appropriate administration (18). Evidence demonstrates that probiotic bacteria exert significant antioxidant effects *in vitro* and *in vivo* (19–22), and the connection between the thyroid gland and gut microbiome is well-established. Furthermore, it has been documented that dysbiosis, an imbalance in gut microbiota, is associated with impaired thyroid function and pathogenesis of thyroid disorders such as Hashimoto’s and Graves’ disease (23). In this review, we discuss the protective role of exogenous nutritional antioxidants in the context of various thyroid disorders. We also review novel findings related to the connection between the thyroid gland and gut microbiome and analyze the effects of probiotics with antioxidant properties on thyroid diseases.

## 2 Search strategy

We searched MEDLINE and PubMed for all English and non-English articles with English abstracts published between 1977 and 2022. The leading search terms were: oxidative stress, reactive oxygen species, thyroid disease, nutritional antioxidants, thyroid-gut axis, gut microbiome, and antioxidant probiotics. The search retrieved original peer-reviewed research articles, which were further analyzed, focusing on the role of nutritional antioxidants in thyroid diseases. We specifically focused on including the most recent findings published in the past five years.

## 3 Oxidative stress

Oxidative stress is a disbalance caused by excessive production of prooxidant substances such as ROS and RNS and/or the antioxidant systems working inefficiently (14, 24, 25). ROS include superoxide anion, hydroxyl radical, and hydrogen peroxide, which are produced *in vivo* primarily by the mitochondrial respiratory chain during aerobic metabolism (26). RNS family includes peroxynitrite, generated *via* a reaction between nitric oxide (NO) and superoxide, and nitrosoperoxycarbonate, generated *via* a reaction between peroxynitrite and carbon dioxide. Under physiological conditions, ROS plays a vital role in maintaining cellular homeostasis by regulating the endogenous antioxidant pool (27–30) and participating in host defence and hormone synthesis (31, 32). In thyrocytes, ROS production is essential for their functional role (33) since TPO-mediated hormone synthesis depends on the action of dual oxidases (DUOX), enzymes responsible for H_2_O_2_ production (34). However, when ROS and RNS are present in excessive amounts and/or in the form of highly reactive free radicals such as superoxide anion and hydroxyl radical, they oxidize susceptible biomolecules such as membrane lipids, cellular proteins, and nucleic acids, leading to disruption of normal cellular functions (35). Lipid peroxidation is a process in which oxidants such as free radicals or non-radical species attack lipids containing carbon-carbon double bond(s), especially polyunsaturated fatty acids (PUFAs) (36). The main lipid peroxidation products are hydroperoxides, such as propanal, hexanal, 4-hydroxynonenal, and malondialdehyde (MDA) (37). Phospholipids, cholesterol, and glycolipids are also targets of potentially lethal peroxidative modifications (38). ROS can also cause damage to DNA by oxidizing nucleoside bases (39). For example, guanine oxidation produces 8-oxo guanine (8-oxoG), which may lead to G-T or G-A transversions if unrepaired. The guanine and deoxyguanosine oxidation products 8-oxoG and its nucleotide 8-oxo-2′-deoxyguanosine (8-oxodG) are ROS-mediated DNA lesions considered the most significant biomarkers for oxidative DNA damage (40). Oxidized bases are usually recognized and repaired by the base excision pathway (BER). Still, when they co-occur on opposing strands, BER can lead to the generation of double-stranded DNA breaks (41). ROS accumulation also induces mitochondrial DNA lesions, strand breaks, and DNA degradation (42). In addition, increased ROS levels are responsible for protein oxidation that can rapidly contribute to the augmentation of OxS by directly affecting cell structure, cell signaling, and essential enzymatic metabolic processes. Several modes of ROS-mediated protein oxidation are reported, including metal-catalyzed oxidation, oxidation-induced cleavage, amino acid oxidation, and the conjugation of lipid peroxidation products (43).

Excessive ROS accumulation is an important factor in the pathogenesis of different diseases. For instance, an elevated ROS production by the respiratory chain is observed in obesity as a response to metabolic overload caused by excess macronutrients and increased substrate availability (44). Mitochondrial dysfunction and endothelial reticulum stress contribute to metabolic perturbances in the adipose tissue of obese patients (45). Consequent ROS accumulation leads to cell damage and pathogenesis of inflammatory and cardiovascular diseases (46). Furthermore, mitochondrial ROS acts as signaling molecules mediating pro-inflammatory cytokines’ production, further reinforcing the connection between OxS and inflammation (47).

Several enzymatic and non-enzymatic defence mechanisms that guard cells against free radical damage have been identified in different cellular localizations, including mitochondria, plasma membrane, endoplasmic reticulum, peroxisomes, and cytosol. For example, enzymes SOD, Cat, and GPx, and transition-metal binding proteins, such as transferrin, ferritin, and ceruloplasmin, inactivate free radicals (48). Three forms of SOD are known in mammals: cytoplasmic SOD (SOD1), mitochondrial SOD (SOD2), and extracellular SOD (SOD3) (49). SOD belongs to a group of metalloenzymes that catalyzes the dismutation of superoxide anion to hydrogen peroxide and molecular oxygen, while Cat decomposes hydrogen peroxide to water and molecular oxygen (50). In high H_2_O_2_ levels, GPx also participates in detoxification by converting lipid peroxides to the corresponding alcohols. Hydrosoluble molecules with free radical scavenging properties such as ascorbic acid, albumin, bilirubin, urates and thiols, liposoluble coenzyme Q10, and vitamin E interfere with the lipid peroxidation by neutralizing the free radicals. In particular, liposoluble scavengers in cellular membranes have high diffusion rates, enabling them to abolish the radical chain reactions by immediately converting them into more stable and less reactive molecules (46). Additional defence mechanisms that reconstruct damaged molecules involve using specific phospholipase that removes peroxidized fatty acids, allowing the reacylation of damaged molecules (51, 52).

## 4 General overview of thyroid diseases

Thyroid hormones have a considerable impact on the cellular oxidative stress processes which is ascribed to their role in cellular metabolism and oxygen consumption (53). Thyroid hormones are produced by thyroid gland, released into circulation, and transported to all organs and cells where they exert their effect. An important role in production of thyroid hormones has hypothalamic-pituitary-thyroid axis. Hypothalamus production of thyrotropin-releasing hormone (TRH) stimulates anterior pituitary gland to secrete thyroid-stimulating hormone (TSH), which affects thyroid gland and leads to production of thyroid hormones. Thyroid gland mainly produces T4, a prohormone which needs to convert to T3 to become biologically active. T4 comprises about 80% of secreted thyroid hormones, while the other 20% is T3. Increased plasma values of thyroid hormones in circulation activate negative feedback loop and inhibit release of TSH (54, 55).

Thyroid hormones exhibit profound metabolic effects characterized by an increased rate of both catabolic and anabolic reactions, resulting in an overall acceleration of the basal metabolism which is associated with increased oxygen consumption, respiratory rate, energy expenditure, and heat production (56). In addition, altered thyroid hormones levels may cause changes in the number and activity of mitochondrial respiratory chain components which represent the principal cellular site of ROS production, ultimately leading to changes in the cellular redox environment and increased ROS generation (57, 58). For instance, it has been reported that hypothyroidism-induced dysfunction of the mitochondrial respiratory chain is associated with increased production of free radicals (59) Thus, excess TSH in hypothyroidism may modulate oxidative stress processes (60) by augmenting the accumulation of ROS that result from both increased generation of free radicals and diminished capacity of the antioxidative defense systems.

Hypothyroidism is associated with an increased risk of atherosclerosis due to its metabolic effects (61, 62). It is commonly accompanied by hyperlipidemia which results from a disbalance between the rates of fatty acids’ synthesis and degradation and is characterized by elevated total cholesterol and low-density lipoprotein-cholesterol (LDL-C), thus providing the substrate for ROS-mediated lipid peroxidation (63–66). Interestingly, products of lipid peroxidation may further increase overall cellular oxidative stress by facilitating the generation of free radicals through the formation of adducts with proteins, which increases direct free radical-induced protein oxidation (67).

In thyroid diseases, metabolic disorders associated with low-grade inflammation can also lead to an increased oxidative stress (68). For instance, chronic low-grade inflammation observed in Hashimoto’s thyroiditis causes endothelial dysfunction which represents an early step in the development of atherosclerosis. Endothelial dysfunction is characterized by the reduction of bioavailability of NO, resulting in impaired endothelium-dependent vasodilation (69) and increased oxidative stress (70). However, it should be mentioned that there is still no consensus in the literature regarding the connection between hypothyroidism and oxidative stress. Some studies report increased oxidative stress in hypothyroidism while other suggest that hypometabolic state that is prevalent in hypothyroidism may protects tissues from oxidative damage.

**Figure 1:**
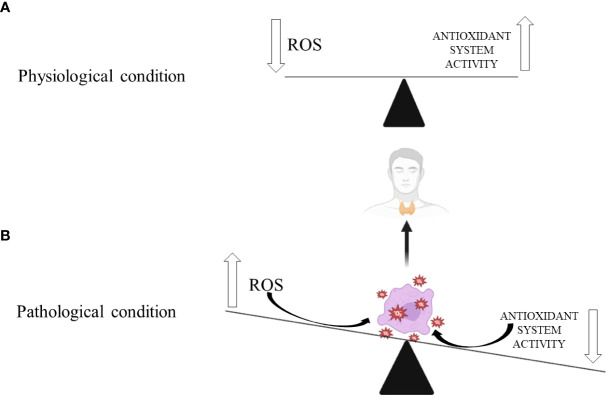
Thyroid gland’s response to oxidative stress in: **(A)** physiological and **(B)** pathological conditions. Biorender.com was used to generate part of the Figure.

Thyroid diseases are considered the most commonly reported endocrine diseases in clinical practice, followed by lipid and carbohydrate disorders (71–73). The primary thyroid condition that affects thyroid functionality presents as hyperthyroidism or hypothyroidism (**Figure 1**.). Thyroid dysfunction could manifest fully (clinically) or latently (subclinically). Based on duration, thyroid dysfunction could be persistent or transitional (73–75). The natural history of thyroid disorders can negatively affect the morphology and function of target tissues if left untreated or improperly treated (55, 73, 76). Thus, the leading causes of death in patients with thyroid dysfunctions are the consequences of atherosclerosis acceleration and the worsening of pre-existing cardiovascular and central nervous system diseases (77, 78). Such endpoints depend on pronounced OxS and diminished antioxidant defense systems at the molecular level (79–81).

## 5 Thyroid diseases and OxS

Different thyroidopathies have been shown to cause increased ROS production and evident OxS-induced damage to thyroid cells. This relationship is reciprocal since thyroid conditions can worsen OxS and increase ROS production, exacerbating oxidative damage. Thyroid hormones increase ROS release in the mitochondrial respiratory chain (25, 82, 83). Hypothyroidism contributes to OxS through an inefficient antioxidant defence system, opposite to hyperthyroidism, where increased ROS production promotes OxS and oxidative damage of thyroid cells (**Figure 1**) (46). According to published research, preventive dietary antioxidant therapy may partially correct the redox imbalance, making it a viable method for preventing the onset of many chronic thyroidopathies (25).

### 5.1 Thyroid dysfunctions and OxS

#### 5.1.1 Hypothyroidism

Even in its subclinical form, hypothyroidism reduces antioxidant system activity, which promotes OxS, causing oxidative damage and altered lipid metabolism in thyroid cells (25, 46, 84). MDA, a by-product of ROS-induced lipid peroxidation, has also been found in higher serum concentrations in hypothyroid patients (79). Even though levothyroxine considerably reduces lipid peroxidation, the serum MDA levels never reach the levels seen in healthy individuals (85). In addition, accumulated oxygen free radicals in thyroid cells may inhibit TPO function and interfere with the synthesis and secretion of thyroid hormones, causing hypothyroidism (34, 86).

#### 5.1.2 Hyperthyroidism

ROS generation is increased by hyperthyroidism (87). The increased intracellular ATP consumption, increased tissue oxygen consumption and oxidative phosphorylation, overexpression of adrenergic receptors, and a decrease in antioxidant defensive mechanisms are the mechanisms of free radicals overproduction that favour OxS in hyperthyroid patients (46). In addition, patients with hyperthyroidism have increased rates of lipid peroxidation compared to euthyroid people, which is a sign of oxidative damage to membrane lipids (82). The link between hyperthyroidism and deteriorating OxS is suggested by the positive association between thyroid hormones and MDA, TSH, and GSH (83).

### 5.2 Thyroid disorders and OxS

#### 5.2.1 Nodular goitre

OxS promotes thyroid cell proliferation (88, 89). Elevated MDA levels were observed in tissues collected from patients with toxic and non-toxic multinodular goitre, accompanied by reduced activity of SOD, GPx, and selenium content compared to adjacent, healthy thyroid tissue. Tissues of benign thyroid nodules show significantly reduced total antioxidant status (TAS) and reduced oxidative stress index (OSI) (90). The presence of elevated OxS parameters in toxic multinodular goitre and decreased plasma GPx and GR activities were also demonstrated (91). These findings suggest an impaired redox balance and antioxidant defence in patients with toxic thyroid nodules and nodular goitre.

Additionally, rare loss-of-function germline mutations of Kelch-like ECH-associated protein 1 (KEAP1) could be detected in nodular goitre leading to Nrf2 pathway activation that favours transcription of cytoprotective and antioxidant enzymes (92). The thyroid nodule size may change in both directions over time. The decrease in the size of thyroid nodules may result from supplementation with extracts of plants with antioxidant and anti-inflammatory properties (93).

#### 5.2.2 Autoimmune thyroid diseases

##### 5.2.2.1 Hashimoto thyroiditis

By interacting with TPO and thyroglobulin (TG) and promoting immunogenicity by altering their morphology and function, NADPH-oxidases (NOXs) involvement in the production of hydrogen peroxide (H_2_O_2_) regarding thyroid hormone synthesis may be related to the pathophysiology of AITD (86, 94, 95). More specifically, it has been demonstrated that an increase in ROS encourages the cleavage of TG into smaller fragments, which exposes the immune system to novel epitopes and intensifies the autoimmune response (96). OxS indicators are significantly higher when Hashimoto thyroiditis is associated with thyroid dysfunction. According to certain studies, the markers of worsened OxS in patients with Hashimoto thyroiditis were closely related to the levels of TG or TPO antibodies (25, 97–99).

Because it increases ROS production and lowers antioxidant levels, excessive iodine consumption is considered an additional risk factor for developing AITD. In people with Hashimoto thyroiditis, anti-TPO antibodies depend on GSH levels and exhibit an inverse correlation (89, 100). Additionally, there is a favourable association between total oxidative status (TOS) and OSI and both antibodies (anti-TG and anti-TPO). Reduced GSH levels seem to be a decisive factor in OxS activation and the development of Hashimoto thyroiditis (101, 102). Additionally, it has been demonstrated that elevated TOS and OSI parameters may precede the development of hypothyroidism in autoimmune thyroiditis and may serve as indicators of thyroid cell injury (101–103). Areas with lower-selenium soil have been linked to increased Hashimoto thyroiditis in humans (104). Also, genetic interactions between minor alleles in the selenoprotein S gene (*SELENOS*) and the nuclear factor erythroid 2-related factor 2 gene (*NFE2L2*) increase chronic thyroiditis incidences (105).

##### 5.2.2.2 Graves’ disease

The most typical cause of hyperthyroidism is Graves’ disease (GD). Its natural history appears to be heavily influenced by oxidative DNA damage. Untreated GD sufferers were shown to have much more DNA damage than patients with toxic nodular goitre and healthy people (106). The highest level of OxS markers was recorded in hyperthyroid GD patients, especially ones with relapsing disease (107, 108). Although both thiamazole and propylthiouracil effectively restore ROS and the antioxidative defence systems, some authors evidenced propylthiouracil as more efficacious (109). The unique mechanism of how OxS leads to GD is disrupting self-tolerance. The thyroid-stimulating antibodies (TSAb) present in GD are engaged in oxidation processes. As the markers of OxS show a positive correlation with TSAb, it may indicate that these variables may be involved in the breakdown of redox balance (110). In patients with GD, activating the nuclear factor erythroid 2–related factor 2 (Nrf2) pathway may help restore thyroid function (105).

#### 5.2.3 Thyroid cancer

Increased production of ROS has been shown to favour cancer development (111). However, ROS can also trigger cell senescence and death, acting as an anti-tumorigenic agent (112). Disturbed genomic integrity induces oxidative genetic damage, DNA oxidation, the activation of proto-oncogenes, and the inactivation tumour suppressor genes leading to proliferative effects and mutagenesis (12, 113, 114). According to Krohn et al., DNA damage, a precursor to tumorigenesis, is thought to be caused by OxS (115). Resultant oxidative DNA base lesions have the potential to mutate some genetic material, which would impair the integrity of the genome by preventing transcription and replication and by generating mutagenesis (116). The oxidized form of guanine, 8-oxo-2′-deoxyguanosine (8-oxo-dG), is a valuable marker of oxidative DNA damage during carcinogenesis (116, 117). When compared to matched normal thyroid tissue, both benign (human follicular adenomas, or FTAs) and malignant (follicular (FTC) and papillary thyroid carcinoma (PTC)) lesions were found to have elevated nuclear levels of 8-oxo-dG (118) which most likely reflects the detrimental effects of prolonged exposure to chronic OxS seen during thyroid cancer (113, 118).

According to an analysis of the redox balance, sera antioxidant levels were lower in thyroid cancer patients than in healthy controls, and OxS marker sera levels in thyroid cancer patients were significantly higher than in the control samples (119). In addition, high concentrations of MDA in blood were detected in thyroid cancer patients, which unequivocally indicated reduced blood antioxidative capacity (120, 121).

A disturbed balance between serum OxS and antioxidant defence system markers is typically encountered in thyroid cancer patients compared to healthy individuals (119, 120, 122, 123). The ineffective defence mechanism cannot neutralize ROS overproduction in thyroid cancer cells, leading to OxS (122). A significant difference in GPx activity and MDA levels was seen between the thyroid cancer patients before and after thyroidectomy in a study examining the change in OxS markers. Although thyroidectomy dramatically improved the oxidative status in favour of antioxidants, lipid peroxidation levels remained much more significant than in healthy thyroid people (25, 121, 124). Also, PTC patients exhibit a worse oxidative profile than patients with autoimmune thyroid disease and higher oxidative process rates than healthy individuals (125). Moreover, thyroid cancer risk was observed to be higher in obese people, and female patients with type 2 diabetes mellitus (T2DM) are more likely to have the extra-glandular invasion of PTC than male T2DM patients (126, 127).

In PTC and anaplastic thyroid carcinoma, somatic *KEAP1* and *NFE2L2* mutations activating the Nrf2 pathway were discovered (128, 129). Although the significance of such pathway activation in thyroid tumours is still unclear, it may help cancer cells survive (105, 130). In addition, tumour tissue exhibit a higher quantity of ROS, which was linked to the decreased expression of selenium antioxidant proteins in cancer cells compared to healthy cells (122). Furthermore, compared to normal thyroid tissue, antioxidant catalase expression was significantly reduced in human thyroid tumours (131). These results show oxidant/antioxidant system in thyroid cancer tissue is imbalanced (12, 132).

## 6 Nutrition and OxS

Proper nutritional intake is mandatory for overall well-being and better human health. However, dietary habits have an impact on human health and can lead to the development of a variety of disorders and diseases. The most prevalent diet in the world is the Western-style diet, characterized by an increased intake of refined food with a high caloric index and an increased amount of sugars and salt, while the intake of vegetables, fruits, and fish is reduced (133). Negative consequences of nutritional habits associated with a Western-style diet may lead to inflammation and production of free radicals (134) through the secretion of numerous pro-inflammatory molecules such as interleukin (IL)- 6, IL-1b, IL-8, and C-reactive protein (CRP), leading to the development of autoimmune disorders either directly, due to inflammation or disturbed immune balance, or indirectly due to increased fat depositions and the development of obesity. Obesity has the most severe consequences since it is associated with systemic inflammation, hypertension, and hypercholesterolemia, which represent conditions that increase the risk of developing cardiovascular disease and T2DM.

It is believed that the state after taking a meal (postprandial state) is pro-inflammatory and pro-oxidative, and the type of food mainly consumed affects the occurrence of OxS. As mentioned earlier, the increased intake of proteins (processed, red meats), sugars, salt, saturated and trans fat, and refined carbohydrates, which are characteristic of the Western-style diet, leads to the development of many diseases, which have their basis in the occurrence of OxS (135). Furthermore, a diet based on an increased intake of carbohydrates and fats leads to increased production of free radicals, directly affecting mitochondrial metabolism. Also, animals fed a high-fat diet have been shown to have increased OxS and dysfunctional mitochondria (136).

To reduce the development of obesity, cancer, diabetes, and cardiovascular diseases, WHO recommended a diet that includes an increased intake of fruits, vegetables, nuts, fish, and unsaturated fatty acids. This type of diet is represented in certain coastal regions of the world and has received the popular name Mediterranean diet. The natural antioxidants, as a result of proper nutritional habits, provide indirect protection by decreasing the production of cytokines and reducing OxS (134).

Numerous exogenous antioxidant molecules (nutritional antioxidants) have been shown to play an important role in excessive ROS accumulation in organisms. Here we will discuss several nutritional antioxidants with a confirmed protective antioxidant role. For instance, monounsaturated fatty acids (MUFA) such as oleic acid that are present in high amounts in olive oil and certain nuts decrease ROS production and exert protection against OxS (137). In addition, oleic acid showed anti-inflammatory effects by decreasing obesity and cytokine production and reducing cardiovascular mortality (134). The anti-inflammatory actions of MUFAs are based on their ability to counteract the effects of long-chain saturated fatty acids on hepatocytes, which include reducing endoplasmic reticulum stress, restricting lipotoxicity induced by accumulation of saturated fatty acids, decreasing ROS production, and inhibiting nuclear factor-κB (NF-kB) transcription factors by binding peroxisome proliferator-activated receptor γ (PPARγ) and G-protein coupled surface receptor 120 (GPR120) (138). *In vitro*, MUFA has shown the ability to induce the expression of the adiponectin gene *via* PPARγ activation, which would result in decreased production of pro-inflammatory molecules such as IL-6 and tumour necrosis factor (TNF)-alpha (139)

Polyunsaturated fatty acids, such as omega-3 fatty acids (n-3 PUFA), are mostly found in eggs, nuts, and fish, whereas omega-6 fatty acid (n-6 PUFA) is predominantly present in sunflower and other vegetable oils. The ratio of n-3 PUFA/n-6 PUFA is of the utmost importance since its disbalance may activate pro-inflammatory pathways (140) n-3 and n-6 PUFA exert opposite effects on the immune system, whereas n-3 PUFA have an anti-inflammatory effect while n-6 PUFA induces a pro-inflammatory action (141). The anti-inflammatory effects of n-3 PUFA are based on their ability to decrease endogenous concentrations of ROS and expression of NF-kB and promote activation of genes involved in antioxidant protection.

Resveratrol (3,4′,5-trihydroxy-trans-stilbene) is a natural polyphenol nonflavonoid compound primarily found in grapes, red wine, berries, and peanuts. It has been shown that long-term treatment with reservatrol prolongs lifespan and reduces OxS (142). In addition, resveratrol was shown to possess cardiovascular protective capacity (143–145) and exhibit antidiabetic, anti-inflammatory, and antioxidant effects (146–150), as well as the ability to suppress the proliferation of a variety of tumour cells (151, 152). Also, resveratrol positively affects obesity, reducing triglycerides and glucose levels. The anti-inflammatory effects resulting from using resveratrol can be seen in the reduction of increased levels of interleukin and TNF in obese mice (15). The antioxidant effects of resveratrol were confirmed in many studies. For example, resveratrol significantly inhibited ROS production by polymorphonuclear leukocytes treated with formyl methionyl leucyl phenylalanine (153) and reduced OxS markers like glycated albumin levels in serum and 8-hydroxyguanosine in urine in stroke-prone spontaneously hypersensitive rats (154). Due to its lipophilic nature, resveratrol can bind to lipoprotein particles, which seems crucial for its antioxidant effects (155). Resveratrol consumption increases plasma antioxidant levels and decreases lipid peroxidation (156). It also reduces intracellular ROS and prevents LDL oxidation in endothelial cells (157) by inhibiting lipoxygenases (158). The mechanism by which resveratrol prevents LDL oxidation is based on its ability to chelate copper and scavenge ROS (159).

Curcumin (1,7-bis(4-hydroxy-3-methoxyphenyl)-1,6-heptadiene-3,5-dione) is a natural polyphenol derived from the rhizomes of the herbs from genus *Curcuma*, particularly from *Curcuma longa* (turmeric), *Curcuma amada, Curcuma zedoaria, Curcuma aromatic* and *Curcuma raktakanta*. (160–162). It has multiple positive effects on the organism, acting as an antioxidant and decreasing inflammation. Using macrophages, Lin et al. showed that curcumin treatment increased levels of SOD and Cat while decreasing levels of ROS (163). The anti-inflammatory effect of curcumin is most likely associated with its ability to inhibit cyclooxygenase-2 (COX-2), lipoxygenase (LOX), and inducible nitric oxide synthase (iNOS) (164). In addition, curcumin positively affects body weight and glucose, increases GPx activity (165), and decreases FFA, triglycerides, and cholesterol concentrations in diabetic rats (15).

Berberine (5,6-dihydro-9,10-dimethoxybenzo [g]-1,3-benzodioxolo [5,6-a] quinolizinium) is a plant alkaloid found and derived from numerous families of plants, such as *Annonaceae, Menispermaceae, Papaveraceae, Ranunculaceae*, etc. (166). Barberine has been shown to have numerous positive effects such as decreasing cholesterol levels and reducing weight and adipose tissue in obese mice. In addition, berberine decreases obesity- and diabetes-related inflammation (167). In the atherosclerotic mouse model, berberine activating the adenosine monophosphate-activated protein kinase (AMPK) signaling pathway decreases OxS (15, 168). Ma et al. (169) showed diabetic animal models administered berberine activates the Nrf2 pathway and decreases OxS.

It is important to mention micronutrients with antioxidant properties, such as vitamins. Since the body cannot synthesize sufficient amounts of vitamins, it is necessary to take them through food or supplements. Vitamin E acts as a regulator of cellular metabolism, and its deficiency leads to anaemia, dysregulation of energy metabolism, irregular mitochondrial function, and tissue damage resulting from increased lipid peroxidation (16). Vitamin D deficiency affects muscle function and leads to irregular cardiovascular function. Also, a lack of Vitamin D is connected with disrupted mitochondrial function and thus increased inflammation and OxS (16). However, it should be emphasized that an increased intake of vitamins has the opposite effect, increasing OxS. For example, it is well known that vitamin C has antioxidant properties; it reacts with ROS creating a product with poor reactivity that does not have detrimental effects. On the other hand, vitamin C may undergo the Fenton reaction, in which a highly reactive free radical is formed (170).

## 7 Nutritional antioxidants and thyroid disease

### 7.1 Trace elements

#### 7.1.1 Iodine

An average adult body contains around 15 to 20 mg of iodine located predominantly in the thyroid gland, which performs an essential role in synthesizing thyroid hormones. In addition to being a component of the thyroid hormone, iodine can act as an antioxidant and antiproliferative agent (171). Its uptake is mediated by the sodium/iodide symporter (NIS), expressed in thyroid cells and extrathyroidal tissues, including the stomach and salivary glands. The iodine content in food is determined by its amount in the soil. Since seafood and seaweed are rich sources of iodine, a diet based on high seafood consumption is sufficient. Likewise, fortifying salt and milk products with iodine ensures an adequate amount of dietary iodine (172, 173). Although iodine deficiency was associated with goitre and thyroid nodules, PTC appears to be more common in areas with high iodine intake, which points to the complex relationship between iodine intake and thyroid disease (174). For instance, excessive iodine intake is associated with a transient reduction of thyroid hormone synthesis for approximately 24 hours after ingestion, known as the Wolff-Chaikoff effect (175). In patients with autoimmune thyroid disease or on anti-thyroid drug therapy, increased iodine intake can induce hypothyroidism, whereas, in patients with diffuse nodular goitre or latent Grave’s disease, it can cause hyperthyroidism (176).

#### 7.1.2 Zinc

Zinc is regarded as an antioxidative trace element because it is a co-factor of the enzyme SOD, which scavenges free radicals. Zinc is essential for normal thyroid function since it is required for the activity of enzyme 1,5′-deiodinase which catalyzes the conversion of T4 to T3. In addition, zinc plays a vital role in the thyroid hormones’ metabolism by regulating thyrotropin-releasing hormone (TRH) and TSH synthesis and modulating the structures of essential transcription factors involved in synthesizing thyroid hormones (177, 178). Zinc deficiency affects the thyroid gland by impairing TRH, TSH, T3, and T4 synthesis. In animal studies, free levels of T3 and T4 were reduced by approximately 30% (179), and a similar trend was observed in studies of human subjects. Hypothyroid patients often present with reduced levels of zinc. In a study designed to evaluate zinc metabolism in patients with thyroid disease, plasma and erythrocyte zinc concentration and urinary zinc excretion were investigated in hypo- and hyperthyroid patients (180). The mean concentration of plasma zinc in hypothyroid patients was lower than that of healthy control subjects, whereas no statistically significant differences were observed in plasma zinc values between hyperthyroid patients and control subjects. However, erythrocyte zinc concentration was significantly decreased in hyperthyroid patients compared to hypothyroid patients and accompanied by an increased urinary zinc excretion resulting from increased muscle tissue catabolism in hyperthyroid patients. The findings of this study suggest that abnormal zinc metabolism commonly occurs in thyroid dysfunctions (180) (**Table 1**).

**Table 1:** The role of nutritional antioxidants in thyroid function.

Nutritional antioxidant	Role in thyroid function	Reference
Iodine	Essential for the synthesis of thyroid hormones Antioxidant Antiproliferative agent	171)
Zinc	Required for the activity of enzyme 1,5′-deiodinase which catalyzes the conversion of T4 to T3 Regulator of TRH and TSH synthesis	(177, 178)
Selenium	Constituent of selenoproteins Cofactor of Gpx, deiodinases and thioredoxin reductases	(181–183)
Resveratrol	Mediates the levels of TSH and iodide uptake in thyrocytes by decreasing sodium/iodide symporter expression	(184)
Berberine	Decreases the abundance of pathogenic bacteria in the gut Increases the content of beneficial bacteria in the gut	(185)
Inositol	Regulates thyroid hormone synthesis by forming H_2_O_2_ in thyrocytes Involved in TSH signaling pathway	(187)
L-carnitine	inhibit thyroid hormone entry into the nucleus of hepatocytes, neurons, and fibroblasts	(186, 187)
Probiotics	*Lactobacilli* and *Bifidobacteriaceae* supplementation increase levothyroxine availability Reduce thyroid hormone serum fluctuation Increase the availability of bacterial enzymes sulfatases and ß-glucuronidases that regulate iodothyronines deconjugation	(188, 189)

TRH, thyrotropin-releasing hormone; TSH, thyroid stimulating hormone; GPx, glutathione peroxidase; H_2_O_2,_ hydrogen peroxide.

#### 7.1.3 Selenium

Selenium is an essential trace mineral whose functions in the organism are mainly connected to its antioxidant properties (190). Selenium is essential to antioxidant enzymes such as GPx (183) and is involved in thyroid and immune system functions. The thyroid gland has the highest concentration of selenium in the body, which is predominantly stored in the thyrocytes in the form of selenoproteins, such as deiodinases, GPx, and thioredoxin reductases (181, 182). Adequate selenium intake is mandatory for the normal function of thyrocytes, and selenium deficiency is associated with the decreased synthesis of thyroid hormones (191), increased thyroid volume, and increased number of thyroid nodules (182, 192). Selenium has been shown to affect T-cell differentiation and modulate the T-helper (Th) cells’ responses. Th cells are cytokine-producing cells that are divided into subgroups 1 and 2 depending on their mechanisms of action; Th1 cells are involved in cell-mediated immunity, whereas Th2 cells participate in antibody-mediated immunity. Th1 cytokine production generally tends to exert pro-inflammatory effects and may lead to autoimmune conditions such as Hashimoto’s thyroiditis. Th2-induced hyperproduction of the thyroid autoantibodies observed in In Graves’ disease results in hyperthyroidism. Selenium deficiency has been associated with Th2 cell response, whereas higher selenium levels favour Th1 response (193). These findings may explain the beneficial effects of selenium supplementation in autoimmune thyroid diseases (181, 194), such as reduced levels of anti-thyroid antibodies, improved thyroid structure and metabolism, and ameliorated clinical symptoms (181, 195). Dietary forms of selenium include selenomethionine present in plant products and inorganic selenium forms used for supplementation (196). No indication of an increased risk of thyroid cancer in either selenium deficiency or exogenous supplementation has been reported (182) (**Table 1**).

### 7.2 Natural polyphenols and alkaloids

As an antioxidant polyphenolic compound and a free radical scavenger, resveratrol has attracted interest for the potential treatment of thyroid diseases accompanied by increased ROS production, such as autoimmune thyroiditis and hyperthyroidism (197). In addition, resveratrol may help treat thyroid cancer since it can induce apoptosis of thyroid cancer cells by increasing the abundance and phosphorylation of p53 tumour suppressor protein (p53) (198, 199). *In vitro* and *in vivo* studies have also demonstrated that resveratrol mediates the levels of TSH and iodide uptake in thyrocytes by decreasing NIS expression (184). However, the observed effects also resulted in significant proliferative action of thyrocytes; thus, resveratrol may be a thyroid-disrupting compound and a goitrogen (184). Currently, data from clinical studies on resveratrol’s effect on the thyroid in humans are absent, and all literature evidence is based on studies performed in cell cultures and animal models. Therefore, proper randomized clinical trials are mandatory to reach the final verdict on the potential use of resveratrol in treating thyroid diseases.

Alkaloid antioxidant berberine was recently reported to exert positive effects in treating GD. When supplemented in combination with methimazole, berberine significantly altered the microbiota composition of patients, decreasing the abundance of the pathogenic bacteria *Chryseobacterium indologenes* and *Enterobacter hormaechei* while simultaneously increasing the content of the beneficial bacteria *Lactococcus lactis (* 185 *)*. In addition, berberine supplementation resulted in significantly elevated enterobactin production, improving iron functioning and restoring thyroid function in patients with Graves’ disease (185) (see **Table 1**).

### 7.3 Inositol

Inositol (also known as vitamin B8) is a carbohydrate compound that is an essential component of the plasma membrane phospholipids and has an important role in synthesizing secondary messengers in the cells (200). Inositol is involved in signaling hormones such as TSH, insulin, and gonadotropins. Myoinositol (Myo), a cyclic polyol with six hydroxyl groups, is the most abundant isoform of inositol, mainly derived from the dietary intake of fruits, beans, and nuts. In contrast, its endogenous production is generated either from glucose by enzymatic reactions or by *de novo* catabolism of phosphatidylinositol (PI), phosphoinositides (PIP), and inositol phosphates (IP). Myo has a crucial role in thyroid function and autoimmune diseases due to its regulation of thyroid hormone synthesis by forming H_2_O_2_ in thyrocytes. Myo is involved in the TSH signaling pathway; thus, depleted levels of Myo may cause the pathogenesis of thyroid diseases such as hypothyroidism (187). It has been observed that TSH levels significantly decreased in patients with subclinical hypothyroidism, with or without autoimmune thyroiditis, after treatment with Myo in combination with selenium (201, 202). Studies of patients with Hashimoto’s thyroiditis and subclinical hypothyroidism showed that supplementation of Myo and selenomethionine significantly decreased TSH, TPOAb, and TGAb concentrations, while simultaneously increasing thyroid hormones levels and restoring euthyroid state in patients with autoimmune thyroiditis (203, 204). In addition, the combined treatment with Myo and selenomethionine was found to have an ameliorating effect on nodular thyroid disease by promoting a significant reduction of thyroid nodules size and number and regression of their stiffness (205). Additional *in vitro* and *in vivo* studies are required to investigate the mechanism of this effect and the potential use of Myo, alone or in combination with selenomethionine, as a novel clinical treatment for the general management of autoimmune thyroiditis and thyroid nodules (**Table 1**).

### 7.4 L-carnitine

L-Carnitine (3-Hydroxy-4-(trimethylazaniumyl) butanoate) is a biological compound that is ubiquitous in mammalian tissues and fluids where it is required for β-oxidation of fatty acids by facilitating their transport in the form of acyl-carnitine esters across the mitochondrial inner membrane (206). In addition, L-carnitine possesses significant antioxidant properties reflected in its ability to scavenge superoxide anion radical and hydrogen peroxide and chelate metal ions such as ferrous ions (207). L-carnitine was shown to positively impact cardiac function through reduced oxidative stress, inflammation, and necrosis of cardiac myocytes (208). As much as 75% of L-carnitine comes from the dietary intake of red meat and dairy products (**Table 2**), whereas only 25% is generated by endogenous biosynthesis. Muscles are the main reservoir of carnitine, storing 95% of the total amount of 120 mmol present in the adult human body (209)

**Table 2:** Classification of nutritional oxidants with protective roles against oxidative stress in thyroid diseases and their naturally occurring sources.

Nutritional antioxidant	Natural source
Vitamins	
Vitamin E	Plant oils (wheat germ, sunflower, safflower, and soybean oil), nuts (almonds, peanuts), sunflower seeds, fruits, and vegetables
Vitamin D	Cod liver oil, salmon, swordfish, tuna fish, sardines, egg yolk, beef liver, dairy and plant milk fortified with vitamin D
Vitamin C	Citrus fruits (oranges, lemon, grapefruit), kiwi, strawberries, vegetables (bell peppers, tomatoes, broccoli, cabbage, cauliflower, white potatoes)
Inositol (vitamin B8)	Fruits (cantaloupe, citrus fruits), fibre-rich foods (beans, brown rice, sesame seeds, corn, wheat bran), nuts (almonds, peanuts)
Trace elements	
Iodine	Seafood, seaweed, iodized table salt, dairy, eggs, chicken, beef liver
Zinc	Seafood, meat
Selenium	Brazil nuts, seafood, meat
Monounsaturated fatty acids (MUFA)	
Oleic acid	Olive and almond oil, nuts (hazelnuts, pecans, almonds)
Polyunsaturated fatty acids (PUFA)	
Omega-3 fatty acids	Seafood, nuts and seeds (flaxseed, chia seeds, and walnuts), plant oils (flaxseed oil, soybean oil, and canola oil)
Omega-6 fatty acids	Vegetable oils (sunflower, corn, and grapeseed oil), nuts (walnuts, pine nuts)
Polyphenolic compounds	
Resveratrol	Grapes, red wine, berries, peanuts
Curcumin	Rhizomes of the herbs from genus *Curcuma* (*Curcuma longa* (turmeric), *Curcuma amada, Curcuma zedoaria, Curcuma aromatic* and *Curcuma raktakanta*)
Alkaloids	
Berberine	Plants (*Annonaceae, Menispermaceae, Papaveraceae, Ranunculaceae*)
Biological compounds	
Carnitine	Red meat, dairy products

The anti-thyroid effect of L-carnitine is based on its ability to inhibit thyroid hormone entry into the nucleus of hepatocytes, neurons, and fibroblasts (186, 210). As a result, rather than being a direct inhibitor of thyroid gland function, it acts as a peripheral antagonist of thyroid hormone action (186). The first controlled clinical trial demonstrating the beneficial effects of L-carnitine in reducing elevated thyroid hormone circulating levels was conducted in 50 women receiving TSH-suppressive (L-T4) therapy for cytologically benign thyroid nodules (210). L-carnitine supplementation was shown to be effective in reversing and preventing symptoms of hyperthyroidism (210). Consequent studies showed that severe forms of GD-related hyperthyroidism, including thyroid storms, may be effectively treated with l-carnitine (211–213), which may be partly explained by increased levels of thyroid hormones deplete the tissue deposits of l-carnitine (214). Interestingly, decreased concentration of L-carnitine was also found in the skeletal muscles of hypothyroid patients (215), suggesting that L-carnitine depletion in skeletal muscles may contribute to myopathy associated with either hypothyroidism or hyperthyroidism. A recent study demonstrated that L-carnitine supplementation might alleviate fatigue symptoms in hypothyroid patients (216). Further clinical studies are required to establish the usefulness of L-carnitine supplementation in hypothyroidism (see **Table 1**).

### 7.5 Probiotics

Probiotics are live non-pathogenic microorganisms with beneficial health effects for their hosts (217). Probiotics regulate the composition of the intestinal microbiota, stimulate humoral and cellular immunity; decrease the frequency and duration of diarrhoea; and eliminate harmful metabolites in the colon, such as ammonium and procancerogenic enzymes. In addition, certain probiotic strains possess antioxidant activity and may reduce damage caused by OxS (218). Probiotics improve metabolic diseases such as obesity and diabetes by modulating intestinal microbiota composition (219–221). Furthermore, the oxidative stress in patients with T2DM can be ameliorated by multispecies probiotics (222).

Probiotic bacteria possess their antioxidant defence systems, such as enzymes SOD and Cat, and can chelate metal ions, such as ferrous and cupric ions, preventing them from catalyzing oxidation (19, 22, 223). In addition, probiotics can stimulate the host’s antioxidant defence systems and increase the activity of antioxidant enzymes (224). For instance, intact cells and cell-free extracts of *Bifidobacterium animalis* 01 can scavenge hydroxyl radicals and superoxide anion *in vitro*, whereas *in vivo*, they increase the antioxidative enzyme activity (20). Lactic acid bacteria strains can defend against peroxide radicals, superoxide anions, and hydroxyl radicals (225, 226). Human studies have shown elevated SOD and GPx activities, and improved total antioxidant status in T2DM patients supplemented with *Lactobacillus acidophilus* La5 and *Bifidobacterium lactis* Bb12 (227). *Lactobacillus rhamnosus* supplementation was shown to exert significant antioxidant protection in conditions of increased physical stress (228). In addition, probiotics produce various antioxidant metabolites, such as GSH and folate. *Lactobacillus fermentum* strains, E-3 and E-18, contain very high levels of GSH (226), which can, together with selenium-dependent GPx, eliminate hydroxyl radicals and peroxynitrite (229).

The enormous complexity of human microbiota is reflected in the finding that adult human organisms typically contain 10^14^ bacteria in the gut, which is approximately ten times more bacterial cells than the number of human cells (230), with at least 400 different bacterial species (231). Most bacterial species in a healthy human microbiota belong to the genera *Bacteroidetes* and *Firmicutes* (232), whereas *Actinobacteria*, *Proteobacteria*, *Fusobacteria*, and *Cyanobacteria*, are less abundant (233). Furthermore, microbiota composition varies depending on its localization in the gastrointestinal system; therefore *Bacilli* class of the *Firmicutes* and *Actinobacteria* is enriched in the small intestine, whereas the *Bacteroidetes* family of the *Firmicutes* is predominantly present in the colon (234).

Gut microbiota in patients with thyroid diseases has a different composition compared to the healthy controls and typically contains a decreased content of *Lactobacillaceae* and *Bifidobacteriaceae.* (23). The family *Lactobacillacae* has important antioxidant properties and may exert protective effects on the thyroid. (235), and its decreased content may cause higher oxidative stress in the thyroid (23). In addition, opportunistic pathogens in gut microbiota were shown in patients suffering from thyroid disease (Zhang, 235). Gut microbiota dysbiosis negatively affects the regulation of anti-inflammatory and immune system responses and appears to be associated with autoimmune diseases, inflammation, and some types of cancer (189, 236, 237). For instance, thyroid cancer is associated with the increased presence of *Clostridiaceae*, *Neisseria*, and *Streptococcus*, whereas in patients with thyroid nodules, a relative increase of *Streptococcus* and *Neisseria* compared to healthy controls was observed (235). Increased abundance of *Neisseria* has been linked to inflammatory disorders (238). In contrast, *Clostridiaceae* and *Streptococcus* were associated with carcinogenic effects and a higher risk of carcinomas (239, 240), and those three seem to have a role in thyroid carcinogenesis (23).

Probiotic supplementation has substantial beneficial effects on thyroid hormones and thyroid function. It was demonstrated that *Lactobacillus reuteri* supplementation improves thyroid function in mice by increasing free T4 and thyroid mass (241). Microbiota modulation by probiotic supplementation of *Lactobacilli* and *Bifidobacteriaceae* increased levothyroxine availability in humans and stabilized thyroid function. Probiotics were shown to be beneficial in lowering serum hormone fluctuations (188), partly because iodothyronines deconjugation is regulated by bacterial enzymes sulfatases and ß-glucuronidases whose availability could be increased by probiotic supplementation (189). Finally, probiotics influence the uptake of minerals relevant to thyroid function, including selenium, iodine, iron, and zinc, and a synergistic effect of probiotics and trace elements on the total antioxidant capacity was observed *in vivo*. Although probiotic supplementation shows promising potential in improving thyroid function in thyroid diseases, further human studies on the effects of probiotics as adjuvant therapy for thyroid diseases are required (**Table 1**).

## 8 Conclusions

Besides the impairment of cellular redox homeostasis in thyroid gland cells, thyroid diseases significantly contribute to systemic redox imbalance. In that way, thyroid diseases are organ-confined and promote histological changes in distant organs by disturbing the vital cellular pathways. Therefore, the simultaneous treatment of thyroid diseases and substituting of nutraceutical antioxidants could beneficially affect different molecular mechanisms enabling the recovery of disturbed redox balance. Se levels are lower in people with thyroid dysfunctions, such as subclinical or overt hypothyroidism (242). In order to determine whether Se supplementation may impact the progression of autoimmune thyroid disease, some trials carried out in regions where the population has a diffusely low or borderline Se status inconsistently suggest that Se supplementation may cause a decrease in thyroid autoantibodies (243, 244). The population heterogeneity, various Se formulations and the length of Se supplementation, as well as different thyroid function test and Se measurement strategies, are among the reasons for the study conclusions inconsistency (244). The benefit of Se supplementation could be expected in patients living in regions with low Se availability or who have low- or sub-optimal Se levels. The supplementation must be attentive as the reference ranges of Se blood levels are narrow, and the risk of insufficient or toxic supplementation is possible (245, 246).

Accumulating evidence support the existence of a thyroid-gut axis and displays important correlations between the composition of the gut bacteria and thyroid function. Dysbiosis, a common finding in thyroid disorders, not only promotes local inflammation of the intestinal membrane but also directly affects thyroid hormone levels *via* its own deiodinase activity and TSH inhibition. In addition, gut microbiota can modulate the absorption of trace minerals, such as iodine, selenium, and zinc, that are essential for thyroid function, including iron. For instance, iodine deficiency may lead to goiter, whereas high iodine intake may induce thyroid dysfunction in susceptible patients. Supplementation with antioxidative probiotics has shown beneficial effects in thyroid diseases thus representing a potential adjuvant therapy for thyroid disorders. The advances in the field of microbiome research envision the future possibility of personalized treatment with probiotics that are specifically adjusted to individual patients. Nevertheless, more data from adequately powered human studies are required for further evaluation of the impact of gut microbiota on thyroid diseases and the potential for possible therapeutic interventions.

## Author contributions

MM wrote the article. ZG wrote the article. SZ wrote the article. ME wrote the article, XG wrote and critically reviewed the article and EI wrote and critically reviewed the article. All authors contributed to the article and approved the submitted version.

## Glossary

**Table:**

8-oxoG	8-oxo guanine
AMPK	adenosine monophosphate-activated protein kinase
BER	base excision repair
CAT	catalase
COX-2	cyclooxygenase-2
CRP	C-reactive protein
GD	Graves’ disease
GPR120	G-protein-coupled surface receptor 120
GPx	glutathione peroxidase
GR	glutathione reductase
GSH	glutathione
H2O2	hydrogen peroxide
iNOS	inducible nitric oxide synthase
IP	inositol phosphates
KEAP1	Kelch-like ECH-associated protein 1
LDL-C	low-density lipoprotein-cholesterol
LOX	lipoxygenase
MDA	malondialdehyde
MUFA	monounsaturated fatty acids
Myo	myoinositol
NFE2L2	nuclear factor erythroid 2-related factor 2 gene
NF-kB	nuclear factor-b
NIS	sodium/iodide symporter
NO	nitric oxide
NOXs	NADPH-oxidases
OSI	oxidative stress index
OxS	oxidative stress
p53	p53 tumour suppressor protein
PI	phosphatidylinositol
PIP	phosphoinositides
PPARγ	peroxisome proliferator-activated receptor γ
PTC	papillary thyroid carcinoma
PUFA	polyunsaturated fatty acids
RNS	reactive nitrogen species
ROS	reactive oxygen species
SELENOS	selenoprotein S gene
SOD	superoxide dismutase
SOD1	cytoplasmic SOD
SOD2	mitochondrial SOD
SOD3	extracellular SOD
T2DM	type 2 diabetes mellitus
T3	triiodothyronine
T4	thyroxine
TAS	total antioxidant status
TG	thyroglobulin
TNF	tumour necrosis factor
TOS	total oxidative status
TPO	thyroid peroxidase
TRH	thyrotropin-releasing hormone
TSAb	thyroid-stimulating antibodies
TSH	thyroid-stimulating hormone

## References

[1] Jakubczyk K Dec K Kałduńska J Kawczuga D Kochman J Janda K. Reactive oxygen species - sources, functions, oxidative damage. Pol Merkur Lekarski (2020) 48:124–7. 32352946

[2] Sies H Jones DP. Reactive oxygen species (ROS) as pleiotropic physiological signalling agents. Nat Rev Mol Cell Biol (2020) 21:363–83. doi: 10.1038/s41580-020-0230-3 32231263

[3] Shekhova E. Mitochondrial reactive oxygen species as major effectors of antimicrobial immunity. PloS Pathog (2020) 16:e1008470. doi: 10.1371/journal.ppat.1008470 32463825 PMC7255592

[4] Yang S Lian G. ROS and diseases: role in metabolism and energy supply. Mol Cell Biochem (2020) 467:1–12. doi: 10.1007/s11010-019-03667-9 31813106 PMC7089381

[5] Di Marzo N Chisci E Giovannoni R. The role of hydrogen peroxide in redox-dependent signaling: Homeostatic and pathological responses in mammalian cells. Cells (2018) 7:156. doi: 10.3390/cells7100156 PMC6211135 30287799

[6] Sies H. Hydrogen peroxide as a central redox signaling molecule in physiological oxidative stress: Oxidative eustress. Redox Biol (2017) 11:613–9. doi: 10.1016/j.redox.2016.12.035 PMC5256672 28110218

[7] Sies H Berndt C Jones DP. Oxidative stress. Annu Rev Biochem (2017) 86:715–48. doi: 10.1146/annurev-biochem-061516-045037 28441057

[8] Schieber M Chandel NS. ROS function in redox signaling and oxidative stress. Curr Biol (2014) 24:R453–462. doi: 10.1016/j.cub.2014.03.034 PMC4055301 24845678

[9] Massart C Hoste C Virion A Ruf J Dumont JE Van Sande J. Cell biology of H2O2 generation in the thyroid: investigation of the control of dual oxidases (DUOX) activity in intact ex vivo thyroid tissue and cell lines. Mol Cell Endocrinol (2011) 343:32–44. doi: 10.1016/j.mce.2011.05.047 21683758

[10] Thanas C Ziros PG Chartoumpekis DV. The Keap1/Nrf2 signaling pathway in the thyroid-2020 update. Antioxidants (Basel) (2020) 9:1082. doi: 10.3390/antiox9111082 PMC7693470 33158045

[11] Poncin S Gérard AC Boucquey M Senou M Calderon PB Knoops B. Oxidative stress in the thyroid gland: from harmlessness to hazard depending on the iodine content. Endocrinology (2008) 149:424–33. doi: 10.1210/en.2007-0951 17884933

[12] Ameziane El Hassani R Buffet C Leboulleux S Dupuy C. Oxidative stress in thyroid carcinomas: biological and clinical significance. Endocr Relat Cancer (2019) 26:R131–r143. doi: 10.1530/erc-18-0476 30615595

[13] Sies H. Oxidative stress: a concept in redox biology and medicine. Redox Biol (2015) 4:180–3. doi: 10.1016/j.redox.2015.01.002 PMC4309861 25588755

[14] Filomeni G De Zio D Cecconi F. Oxidative stress and autophagy: the clash between damage and metabolic needs. Cell Death Differ (2015) 22:377–88. doi: 10.1038/cdd.2014.150 PMC4326572 25257172

[15] Gu Y Han J Jiang C Zhang Y. Biomarkers, oxidative stress and autophagy in skin aging. Ageing Res Rev (2020) 59:101036. doi: 10.1016/j.arr.2020.101036 32105850

[16] Margaritelis NV Paschalis V Theodorou AA Kyparos A Nikolaidis MG. Antioxidants in personalized nutrition and exercise. Adv Nutr (2018) 9:813–23. doi: 10.1093/advances/nmy052 PMC6247356 30256898

[17] Guo Q Li F Duan Y Wen C Wang W Zhang L. Oxidative stress, nutritional antioxidants and beyond. Sci China Life Sci (2020) 63:866–74. doi: 10.1007/s11427-019-9591-5 31705360

[18] Williams NT. Probiotics. Am J Health Syst Pharm (2010) 67:449–58. doi: 10.2146/ajhp090168 20208051

[19] Lin MY Yen CL. Antioxidative ability of lactic acid bacteria. J Agric Food Chem (1999) 47:1460–6. doi: 10.1021/jf981149l 10563999

[20] Shen Q Shang N Li P. *In vitro* and *in vivo* antioxidant activity of bifidobacterium animalis 01 isolated from centenarians. Curr Microbiol (2011) 62:1097–103. doi: 10.1007/s00284-010-9827-7 21132298

[21] Persichetti E De Michele A Codini M Traina G. Antioxidative capacity of lactobacillus fermentum LF31 evaluated *in vitro* by oxygen radical absorbance capacity assay. Nutrition (2014) 30:936–8. doi: 10.1016/j.nut.2013.12.009 24985014

[22] Wang Y Wu Y Wang Y Fu A Gong L Li W. Bacillus amyloliquefaciens SC06 alleviates the oxidative stress of IPEC-1 *via* modulating Nrf2/Keap1 signaling pathway and decreasing ROS production. Appl Microbiol Biotechnol (2017) 101:3015–26. doi: 10.1007/s00253-016-8032-4 27957629

[23] Knezevic J Starchl C Tmava Berisha A Amrein K. Thyroid-Gut-Axis: How does the microbiota influence thyroid function? Nutrients (2020) 12:1769. doi: 10.3390/nu12061769 32545596 PMC7353203

[24] Marrocco I Altieri F. Measurement and clinical significance of biomarkers of oxidative stress in humans. Oxid Med Cell Longev (2017) 2017:6501046. doi: 10.1155/2017/6501046 28698768 PMC5494111

[25] Kochman J Jakubczyk K. The influence of oxidative stress on thyroid diseases. Antioxidants (Basel) (2021) 10:1442. doi: 10.3390/antiox10091442 34573074 PMC8465820

[26] Kang D Hamasaki N. Mitochondrial oxidative stress and mitochondrial DNA. Clin Chem Lab Med (2003) 41:1281–8. doi: 10.1515/cclm.2003.195 14580153

[27] Le Bras M Clément MV Pervaiz S Brenner C. Reactive oxygen species and the mitochondrial signaling pathway of cell death. Histol Histopathol (2005) 20:205–19. doi: 10.14670/hh-20.205 15578439

[28] Valko M Leibfritz D Moncol J Cronin MT Mazur M Telser J. Free radicals and antioxidants in normal physiological functions and human disease. Int J Biochem Cell Biol (2007) 39:44–84. doi: 10.1016/j.biocel.2006.07.001 16978905

[29] Wink DA Hines HB Cheng RY Switzer CH Flores-Santana W Vitek MP. Nitric oxide and redox mechanisms in the immune response. J Leukoc Biol (2011) 89:873–91. doi: 10.1189/jlb.1010550 PMC3100761 21233414

[30] Mangge H Becker K Fuchs D Gostner JM. Antioxidants, inflammation and cardiovascular disease. World J Cardiol (2014) 6:462–77. doi: 10.4330/wjc.v6.i6.462 PMC4072837 24976919

[31] Lambeth JD. NOX enzymes and the biology of reactive oxygen. Nat Rev Immunol (2004) 4:181–9. doi: 10.1038/nri1312 15039755

[32] Quinn MT Gauss KA. Structure and regulation of the neutrophil respiratory burst oxidase: comparison with nonphagocyte oxidases. J Leukoc Biol (2004) 76:760–81. doi: 10.1189/jlb.0404216 15240752

[33] Cross AR Jones OT. Enzymic mechanisms of superoxide production. Biochim Biophys Acta (1991) 1057:281–98. doi: 10.1016/s0005-2728(05)80140-9 1851438

[34] Ohye H Sugawara M. Dual oxidase, hydrogen peroxide and thyroid diseases. Exp Biol Med (Maywood) (2010) 235:424–33. doi: 10.1258/ebm.2009.009241 20407074

[35] Monaghan P Metcalfe NB Torres R. Oxidative stress as a mediator of life history trade-offs: mechanisms, measurements and interpretation. Ecol Lett (2009) 12:75–92. doi: 10.1111/j.1461-0248.2008.01258.x 19016828

[36] Yin H Xu L Porter NA. Free radical lipid peroxidation: mechanisms and analysis. Chem Rev (2011) 111:5944–72. doi: 10.1021/cr200084z 21861450

[37] Yoshida Y Umeno A Shichiri M. Lipid peroxidation biomarkers for evaluating oxidative stress and assessing antioxidant capacity *in vivo* . J Clin Biochem Nutr (2013) 52:9–16. doi: 10.3164/jcbn.12-112 23341691 PMC3541426

[38] Ayala A Muñoz MF Argüelles S. Lipid peroxidation: production, metabolism, and signaling mechanisms of malondialdehyde and 4-hydroxy-2-nonenal. Oxid Med Cell Longev (2014) 2014:360438. doi: 10.1155/2014/360438 24999379 PMC4066722

[39] Cooke MS Evans MD Dizdaroglu M Lunec J. Oxidative DNA damage: mechanisms, mutation, and disease. FASEB J (2003) 17:1195–214. doi: 10.1096/fj.02-0752rev 12832285

[40] Chiorcea-Paquim AM. 8-oxoguanine and 8-oxodeoxyguanosine biomarkers of oxidative DNA damage: A review on HPLC-ECD determination. Molecules (2022) 27:1620. doi: 10.3390/molecules27051620 35268721 PMC8911600

[41] Cannan WJ Tsang BP Wallace SS Pederson DS. Nucleosomes suppress the formation of double-strand DNA breaks during attempted base excision repair of clustered oxidative damages. J Biol Chem (2014) 289:19881–93. doi: 10.1074/jbc.M114.571588 PMC4106309 24891506

[42] Shokolenko I Venediktova N Bochkareva A Wilson GL Alexeyev MF. Oxidative stress induces degradation of mitochondrial DNA. Nucleic Acids Res (2009) 37:2539–48. doi: 10.1093/nar/gkp100 PMC2677867 19264794

[43] Cecarini V Gee J Fioretti E Amici M Angeletti M Eleuteri AM. Protein oxidation and cellular homeostasis: Emphasis on metabolism. Biochim Biophys Acta (2007) 1773:93–104. doi: 10.1016/j.bbamcr.2006.08.039 17023064

[44] Turrens JF Boveris A. Generation of superoxide anion by the NADH dehydrogenase of bovine heart mitochondria. Biochem J (1980) 191:421–7. doi: 10.1042/bj1910421 PMC1162232 6263247

[45] Zimmermann MB Aeberli I. Dietary determinants of subclinical inflammation, dyslipidemia and components of the metabolic syndrome in overweight children: a review. Int J Obes (Lond) (2008) 32 Suppl 6:S11–18. doi: 10.1038/ijo.2008.202 19079275

[46] Mancini A Di Segni C. Thyroid hormones, oxidative stress, and inflammation. Mediators Inflamm (2016) 2016:6757154. doi: 10.1155/2016/6757154 27051079 PMC4802023

[47] Siti HN Kamisah Y Kamsiah J. The role of oxidative stress, antioxidants and vascular inflammation in cardiovascular disease (a review). Vascul Pharmacol (2015) 71:40–56. doi: 10.1016/j.vph.2015.03.005 25869516

[48] Inoue K Sakano N Ogino K Sato Y Wang DH Kubo M. Relationship between ceruloplasmin and oxidative biomarkers including ferritin among healthy Japanese. J Clin Biochem Nutr (2013) 52:160–6. doi: 10.3164/jcbn.12-122 PMC3593134 23524455

[49] Kim SH Kim SH Lee JH Lee BH Yoon HJ Shin DH. Superoxide dismutase gene (SOD1, SOD2, and SOD3) polymorphisms and antituberculosis drug-induced hepatitis. Allergy Asthma Immunol Res (2015) 7:88–91. doi: 10.4168/aair.2015.7.1.88 25553268 PMC4274475

[50] Yasui K Baba A. Therapeutic potential of superoxide dismutase (SOD) for resolution of inflammation. Inflammation Res (2006) 55:359–63. doi: 10.1007/s00011-006-5195-y 17122956

[51] Nigam S Schewe T. Phospholipase A(2)s and lipid peroxidation. Biochim Biophys Acta (2000) 1488:167–81. doi: 10.1016/s1388-1981(00)00119-0 11080686

[52] Six DA Dennis EA. The expanding superfamily of phospholipase A(2) enzymes: classification and characterization. Biochim Biophys Acta (2000) 1488:1–19. doi: 10.1016/s1388-1981(00)00105-0 11080672

[53] Messarah M Saoudi M Boumendjel A Boulakoud MS Feki AE. Oxidative stress induced by thyroid dysfunction in rat erythrocytes and heart. Environ Toxicol Pharmacol (2011) 31:33–41. doi: 10.1016/j.etap.2010.09.003 21787667

[54] van der Spek AH Fliers E Boelen A. Thyroid hormone metabolism in innate immune cells. J Endocrinol (2017) 232:R67–r81. doi: 10.1530/joe-16-0462 27852725

[55] Gluvic ZM Zafirovic SS Obradovic MM Sudar-Milovanovic EM Rizzo M Isenovic ER. Hypothyroidism and risk of cardiovascular disease. Curr Pharm Des (2022) 28:2065–72. doi: 10.2174/1381612828666220620160516 35726428

[56] Chakrabarti SK Ghosh S Banerjee S Mukherjee S Chowdhury S. Oxidative stress in hypothyroid patients and the role of antioxidant supplementation. Indian J Endocrinol Metab (2016) 20:674–8. doi: 10.4103/2230-8210.190555 PMC5040049 27730079

[57] Venditti P Di Meo S. Thyroid hormone-induced oxidative stress. Cell Mol Life Sci (2006) 63:414–34. doi: 10.1007/s00018-005-5457-9 PMC11136030 16389448

[58] Chattopadhyay S Sahoo DK Roy A Samanta L Chainy GB. Thiol redox status critically influences mitochondrial response to thyroid hormone-induced hepatic oxidative injury: A temporal analysis. Cell Biochem Funct (2010) 28:126–34. doi: 10.1002/cbf.1631 20087846

[59] Resch U Helsel G Tatzber F Sinzinger H. Antioxidant status in thyroid dysfunction. Clin Chem Lab Med (2002) 40:1132–4. doi: 10.1515/cclm.2002.198 12521231

[60] Nanda N Bobby Z Hamide A. Association of thyroid stimulating hormone and coronary lipid risk factors with lipid peroxidation in hypothyroidism. Clin Chem Lab Med (2008) 46:674–9. doi: 10.1515/cclm.2008.139 18598205

[61] Hak AE Pols HA Visser TJ Drexhage HA Hofman A Witteman JC. Subclinical hypothyroidism is an independent risk factor for atherosclerosis and myocardial infarction in elderly women: the Rotterdam study. Ann Intern Med (2000) 132:270–8. doi: 10.7326/0003-4819-132-4-200002150-00004 10681281

[62] Gluvic Z Sudar E Tica J Jovanovic A Zafirovic S Tomasevic R. Effects of levothyroxine replacement therapy on parameters of metabolic syndrome and atherosclerosis in hypothyroid patients: a prospective pilot study. Int J Endocrinol (2015) 2015:147070. doi: 10.1155/2015/147070 25821465 PMC4363579

[63] Duntas LH. Thyroid disease and lipids. Thyroid (2002) 12:287–93. doi: 10.1089/10507250252949405 12034052

[64] Fernández V Tapia G Varela P Romanque P Cartier-Ugarte D Videla LA. Thyroid hormone-induced oxidative stress in rodents and humans: a comparative view and relation to redox regulation of gene expression. Comp Biochem Physiol C Toxicol Pharmacol (2006) 142:231–9. doi: 10.1016/j.cbpc.2005.10.007 16298169

[65] Peppa M Betsi G Dimitriadis G. Lipid abnormalities and cardiometabolic risk in patients with overt and subclinical thyroid disease. J Lipids (2011) 2011:575840. doi: 10.1155/2011/575840 21789282 PMC3140027

[66] Rizos CV Elisaf MS Liberopoulos EN. Effects of thyroid dysfunction on lipid profile. Open Cardiovasc Med J (2011) 5:76–84. doi: 10.2174/1874192401105010076 21660244 PMC3109527

[67] Negre-Salvayre A Coatrieux C Ingueneau C Salvayre R. Advanced lipid peroxidation end products in oxidative damage to proteins. potential role in diseases and therapeutic prospects for the inhibitors. Br J Pharmacol (2008) 153:6–20. doi: 10.1038/sj.bjp.0707395 17643134 PMC2199390

[68] Gluvic ZM Obradovic MM Sudar-Milovanovic EM Zafirovic SS Radak DJ Essack MM. Regulation of nitric oxide production in hypothyroidism. BioMed Pharmacother (2020) 124:109881. doi: 10.1016/j.biopha.2020.109881 31986413

[69] Lerman A Burnett JC Jr. Intact and altered endothelium in regulation of vasomotion. Circulation (1992) 86:III12–19. 1424046

[70] Taddei S Caraccio N Virdis A Dardano A Versari D Ghiadoni L. Low-grade systemic inflammation causes endothelial dysfunction in patients with hashimoto’s thyroiditis. J Clin Endocrinol Metab (2006) 91:5076–82. doi: 10.1210/jc.2006-1075 16968790

[71] Canaris GJ Manowitz NR Mayor G Ridgway EC. The Colorado thyroid disease prevalence study. Arch Intern Med (2000) 160:526–34. doi: 10.1001/archinte.160.4.526 10695693

[72] Hollowell JG Staehling NW Flanders WD Hannon WH Gunter EW Spencer CA. Serum TSH, T(4), and thyroid antibodies in the united states population, (1988 to 1994): National health and nutrition examination survey (NHANES III). J Clin Endocrinol Metab (2002) 87:489–99. doi: 10.1210/jcem.87.2.8182 11836274

[73] Berta E Lengyel I Halmi S Zrínyi M Erdei A Harangi M. Hypertension in thyroid disorders. Front Endocrinol (Lausanne) (2019) 10:482. doi: 10.3389/fendo.2019.00482 31379748 PMC6652798

[74] De Leo S Lee SY Braverman LE. Hyperthyroidism. Lancet (2016) 388:906–18. doi: 10.1016/s0140-6736(16)00278-6 PMC5014602 27038492

[75] Chaker L Bianco AC Jonklaas J Peeters RP. Hypothyroidism. Lancet (2017) 390:1550–62. doi: 10.1016/s0140-6736(17)30703-1 PMC6619426 28336049

[76] Delitala AP. Subclinical hyperthyroidism and the cardiovascular disease. Horm Metab Res (2017) 49:723–31. doi: 10.1055/s-0043-117893 28915531

[77] Stamatouli A Bedoya P Yavuz S. Hypothyroidism: Cardiovascular endpoints of thyroid hormone replacement. Front Endocrinol (Lausanne) (2019) 10:888. doi: 10.3389/fendo.2019.00888 31998229 PMC6962138

[78] Barreiro Arcos ML. Role of thyroid hormones-induced oxidative stress on cardiovascular physiology. Biochim Biophys Acta Gen Subj (2022) 1866:130239. doi: 10.1016/j.bbagen.2022.130239 36064072

[79] Erdamar H Cimen B Gülcemal H Saraymen R Yerer B Demirci H. Increased lipid peroxidation and impaired enzymatic antioxidant defense mechanism in thyroid tissue with multinodular goiter and papillary carcinoma. Clin Biochem (2010) 43:650–4. doi: 10.1016/j.clinbiochem.2010.02.005 20171198

[80] Ramli NSF Mat Junit S Leong NK Razali N. Analyses of antioxidant status and nucleotide alterations in genes encoding antioxidant enzymes in patients with benign and malignant thyroid disorders. PeerJ (2017) 5:. doi: 10.7717/peerj.3365 PMC5457668 28584708

[81] Kuzan A Królewicz E. Contribution of glycation and oxidative stress to thyroid gland pathology-a pilot study. Biomolecules (2021) 11:557. doi: 10.3390/biom11040557 33920190 PMC8069218

[82] Piazera BKL Gomes DV Vigário P Salerno VP Vaisman M. Evaluation of redox profiles in exogenous subclinical hyperthyroidism at two different levels of TSH suppression. Arch Endocrinol Metab (2018) 62:545–51. doi: 10.20945/2359-3997000000075 PMC10118655 30462808

[83] Fahim YA Sharaf NE. Assessment of thyroid function and oxidative stress state in foundry workers exposed to lead. J Health Pollut (2020) 10:200903. doi: 10.5696/2156-9614-10.27.200903 32874759 PMC7453815

[84] Torun AN Kulaksizoglu S Kulaksizoglu M Pamuk BO Isbilen E Tutuncu NB. Serum total antioxidant status and lipid peroxidation marker malondialdehyde levels in overt and subclinical hypothyroidism. Clin Endocrinol (Oxf) (2009) 70:469–74. doi: 10.1111/j.1365-2265.2008.03348.x 18727709

[85] Baskol G Atmaca H Tanriverdi F Baskol M Kocer D Bayram F. Oxidative stress and enzymatic antioxidant status in patients with hypothyroidism before and after treatment. Exp Clin Endocrinol Diabetes (2007) 115:522–6. doi: 10.1055/s-2007-981457 17853336

[86] Fortunato RS Ferreira AC Hecht F Dupuy C Carvalho DP. Sexual dimorphism and thyroid dysfunction: a matter of oxidative stress? J Endocrinol (2014) 221:R31–40. doi: 10.1530/joe-13-0588 24578296

[87] Marcocci C Bartalena L. Role of oxidative stress and selenium in graves’ hyperthyroidism and orbitopathy. J Endocrinol Invest (2013) 36:15–20. 24419055

[88] Poncin S Van Eeckoudt S Humblet K Colin IM Gérard AC. Oxidative stress: a required condition for thyroid cell proliferation. Am J Pathol (2010) 176:1355–63. doi: 10.2353/ajpath.2010.090682 PMC2832155 20093493

[89] Ruggeri RM Giovinazzo S Barbalace MC Cristani M Alibrandi A Vicchio TM. Influence of dietary habits on oxidative stress markers in hashimoto’s thyroiditis. Thyroid (2021) 31:96–105. doi: 10.1089/thy.2020.0299 32729374

[90] Faam B Ghadiri AA Ghaffari MA Totonchi M Khorsandi L. Comparing oxidative stress status among Iranian males and females with malignant and non-malignant thyroid nodules. Int J Endocrinol Metab (2021) 19:e105669. doi: 10.5812/ijem.105669 33815516 PMC8010567

[91] Bednarek J Wysocki H Sowinski J. Oxidation products and antioxidant markers in plasma of patients with graves’ disease and toxic multinodular goiter: effect of methimazole treatment. Free Radic Res (2004) 38:659–64. doi: 10.1080/10715760410001701621 15346657

[92] Nishihara E Hishinuma A Kogai T Takada N Hirokawa M Fukata S. A novel germline mutation of KEAP1 (R483H) associated with a non-toxic multinodular goiter. Front Endocrinol (Lausanne) (2016) 7:131. doi: 10.3389/fendo.2016.00131 27703446 PMC5028897

[93] Stancioiu F Mihai D Papadakis GZ Tsatsakis A Spandidos DA Badiu C. Treatment for benign thyroid nodules with a combination of natural extracts. Mol Med Rep (2019) 20:2332–8. doi: 10.3892/mmr.2019.10453 PMC6691239 31322200

[94] Duthoit C Estienne V Giraud A Durand-Gorde JM Rasmussen AK Feldt-Rasmussen U. Hydrogen peroxide-induced production of a 40 kDa immunoreactive thyroglobulin fragment in human thyroid cells: the onset of thyroid autoimmunity? Biochem J (2001) 360:557–62. doi: 10.1042/0264-6021:3600557 PMC1222257 11736644

[95] Gheorghiu ML Badiu C. Selenium involvement in mitochondrial function in thyroid disorders. Hormones (Athens) (2020) 19:25–30. doi: 10.1007/s42000-020-00173-2 31960358

[96] Niethammer P Grabher C Look AT Mitchison TJ. A tissue-scale gradient of hydrogen peroxide mediates rapid wound detection in zebrafish. Nature (2009) 459:996–9. doi: 10.1038/nature08119 PMC2803098 19494811

[97] Ruggeri RM Vicchio TM Cristani M Certo R Caccamo D Alibrandi A. Oxidative stress and advanced glycation end products in hashimoto’s thyroiditis. Thyroid (2016) 26:504–11. doi: 10.1089/thy.2015.0592 26854840

[98] Li D Liang G Calderone R Bellanti JA. Vitiligo and hashimoto’s thyroiditis: Autoimmune diseases linked by clinical presentation, biochemical commonality, and autoimmune/oxidative stress-mediated toxicity pathogenesis. Med Hypotheses (2019) 128:69–75. doi: 10.1016/j.mehy.2019.05.010 31203913

[99] Ruggeri RM CampennÌ A Giuffrida G Casciaro M Barbalace MC Hrelia S. Oxidative stress as a key feature of autoimmune thyroiditis: an update. Minerva Endocrinol (2020) 45:326–44. doi: 10.23736/s0391-1977.20.03268-x 32969631

[100] Rostami R Nourooz-Zadeh S Mohammadi A Khalkhali HR Ferns G Nourooz-Zadeh J. Serum selenium status and its interrelationship with serum biomarkers of thyroid function and antioxidant defense in hashimoto’s thyroiditis. Antioxidants (Basel) (2020) 9:1070. doi: 10.3390/antiox9111070 33142736 PMC7692168

[101] Rostami R Aghasi MR Mohammadi A Nourooz-Zadeh J. Enhanced oxidative stress in hashimoto’s thyroiditis: inter-relationships to biomarkers of thyroid function. Clin Biochem (2013) 46:308–12. doi: 10.1016/j.clinbiochem.2012.11.021 23219737

[102] Ates I Arikan MF Altay M Yilmaz FM Yilmaz N Berker D. The effect of oxidative stress on the progression of hashimoto’s thyroiditis. Arch Physiol Biochem (2018) 124:351–6. doi: 10.1080/13813455.2017.1408660 29185364

[103] Baser H Can U Baser S Yerlikaya FH Aslan U Hidayetoglu BT. Assesment of oxidative status and its association with thyroid autoantibodies in patients with euthyroid autoimmune thyroiditis. Endocrine (2015) 48:916–23. doi: 10.1007/s12020-014-0399-3 25150037

[104] Valea A Georgescu CE. Selenoproteins in human body: focus on thyroid pathophysiology. Hormones (Athens) (2018) 17:183–96. doi: 10.1007/s42000-018-0033-5 29873029

[105] Chartoumpekis DV Ziros PG Habeos IG Sykiotis GP. Emerging roles of Keap1/Nrf2 signaling in the thyroid gland and perspectives for bench-to-bedside translation. Free Radic Biol Med (2022) 190:276–83. doi: 10.1016/j.freeradbiomed.2022.08.021 35988853

[106] Zarković M. The role of oxidative stress on the pathogenesis of graves’ disease. J Thyroid Res (2012) 2012:302537. doi: 10.1155/2012/302537 22175033 PMC3235898

[107] Ademoğlu E Ozbey N Erbil Y Tanrikulu S Barbaros U Yanik BT. Determination of oxidative stress in thyroid tissue and plasma of patients with graves’ disease. Eur J Intern Med (2006) 17:545–50. doi: 10.1016/j.ejim.2006.04.013 17142172

[108] Maouche N Meskine D Alamir B Koceir EA. Trace elements profile is associated with insulin resistance syndrome and oxidative damage in thyroid disorders: Manganese and selenium interest in Algerian participants with dysthyroidism. J Trace Elem Med Biol (2015) 32:112–21. doi: 10.1016/j.jtemb.2015.07.002 26302919

[109] Kocak M Akarsu E Korkmaz H Taysi S. The effect of antithyroid drugs on osteopontin and oxidative stress in graves’ disease. Acta Endocrinol (Buchar) (2019) 15:221–4. doi: 10.4183/aeb.2019.221 PMC6711651 31508180

[110] Diana T Daiber A Oelze M Neumann S Olivo PD Kanitz M. Stimulatory TSH-receptor antibodies and oxidative stress in graves disease. J Clin Endocrinol Metab (2018) 103:3668–77. doi: 10.1210/jc.2018-00509 PMC6179174 30099546

[111] Valko M Rhodes CJ Moncol J Izakovic M Mazur M. Free radicals, metals and antioxidants in oxidative stress-induced cancer. Chem Biol Interact (2006) 160:1–40. doi: 10.1016/j.cbi.2005.12.009 16430879

[112] Collery P. Strategies for the development of selenium-based anticancer drugs. J Trace Elem Med Biol (2018) 50:498–507. doi: 10.1016/j.jtemb.2018.02.024 29548612

[113] Stone JR. An assessment of proposed mechanisms for sensing hydrogen peroxide in mammalian systems. Arch Biochem Biophys (2004) 422:119–24. doi: 10.1016/j.abb.2003.12.029 14759598

[114] Nakashima M Suzuki K Meirmanov S Naruke Y Matsuu-Matsuyama M Shichijo K. Foci formation of P53-binding protein 1 in thyroid tumors: activation of genomic instability during thyroid carcinogenesis. Int J Cancer (2008) 122:1082–8. doi: 10.1002/ijc.23223 17985346

[115] Krohn K Maier J Paschke R. Mechanisms of disease: hydrogen peroxide, DNA damage and mutagenesis in the development of thyroid tumors. Nat Clin Pract Endocrinol Metab (2007) 3:713–20. doi: 10.1038/ncpendmet0621 17893690

[116] Sedelnikova OA Redon CE Dickey JS Nakamura AJ Georgakilas AG Bonner WM. Role of oxidatively induced DNA lesions in human pathogenesis. Mutat Res (2010) 704:152–9. doi: 10.1016/j.mrrev.2009.12.005 PMC3074954 20060490

[117] Kasai H. Analysis of a form of oxidative DNA damage, 8-hydroxy-2’-deoxyguanosine, as a marker of cellular oxidative stress during carcinogenesis. Mutat Res (1997) 387:147–63. doi: 10.1016/s1383-5742(97)00035-5 9439711

[118] Karger S Krause K Engelhardt C Weidinger C Gimm O Dralle H. Distinct pattern of oxidative DNA damage and DNA repair in follicular thyroid tumours. J Mol Endocrinol (2012) 48:193–202. doi: 10.1530/jme-11-0119 22331172

[119] Wang D Feng JF Zeng P Yang YH Luo J Yang YW. Total oxidant/antioxidant status in sera of patients with thyroid cancers. Endocr Relat Cancer (2011) 18:773–82. doi: 10.1530/erc-11-0230 PMC3230112 22002574

[120] Gerić M Domijan AM Gluščić V Janušić R Šarčević B Garaj-Vrhovac V. Cytogenetic status and oxidative stress parameters in patients with thyroid diseases. Mutat Res Genet Toxicol Environ Mutagen (2016) 810:22–9. doi: 10.1016/j.mrgentox.2016.09.010 27776688

[121] Heydarzadeh S Kia SK. The cross-talk between polyphenols and the target enzymes related to oxidative stress-induced thyroid cancer. Oxid Med Cell Longev (2022) 2022:2724324. doi: 10.1155/2022/2724324 35571253 PMC9098327

[122] Metere A Frezzotti F Graves CE Vergine M De Luca A Pietraforte D. A possible role for selenoprotein glutathione peroxidase (GPx1) and thioredoxin reductases (TrxR1) in thyroid cancer: our experience in thyroid surgery. Cancer Cell Int (2018) 18:7. doi: 10.1186/s12935-018-0504-4 29371830 PMC5769232

[123] Rovcanin B Stojsavljevic A Kekic D Gopcevic K Manojlovic D Jovanovic M. Redox status and antioxidative cofactor metals influence clinical and pathological characteristics of papillary thyroid carcinoma and colloid goiter. Biol Trace Elem Res (2020) 197:349–59. doi: 10.1007/s12011-019-01995-x 31811573

[124] Akinci M Kosova F Cetin B Sepici A Altan N Aslan S. Oxidant/antioxidant balance in patients with thyroid cancer. Acta Cir Bras (2008) 23:551–4. doi: 10.1590/s0102-86502008000600013 19030755

[125] Lassoued S Mseddi M Mnif F Abid M Guermazi F Masmoudi H. A comparative study of the oxidative profile in graves’ disease, hashimoto’s thyroiditis, and papillary thyroid cancer. Biol Trace Elem Res (2010) 138:107–15. doi: 10.1007/s12011-010-8625-1 20204550

[126] Matrone A Ferrari F Santini F Elisei R. Obesity as a risk factor for thyroid cancer. Curr Opin Endocrinol Diabetes Obes (2020) 27:358–63. doi: 10.1097/med.0000000000000556 32740043

[127] Shi P Zhang L Liu Y Yang F Fu K Li R. Clinicopathological features and prognosis of papillary thyroid cancer patients with type 2 diabetes mellitus. Gland Surg (2022) 11:358–68. doi: 10.21037/gs-21-905 PMC8899419 35284317

[128] Ziros PG Habeos IG Chartoumpekis DV Ntalampyra E Somm E Renaud CO. NFE2-related transcription factor 2 coordinates antioxidant defense with thyroglobulin production and iodination in the thyroid gland. Thyroid (2018) 28:780–98. doi: 10.1089/thy.2018.0018 PMC5994681 29742982

[129] Renaud CO Ziros PG Chartoumpekis DV Bongiovanni M Sykiotis GP. Keap1/Nrf2 signaling: A new player in thyroid pathophysiology and thyroid cancer. Front Endocrinol (Lausanne) (2019) 10:510. doi: 10.3389/fendo.2019.00510 31428048 PMC6687762

[130] Leinonen HM Kansanen E Pölönen P Heinäniemi M Levonen AL. Role of the Keap1-Nrf2 pathway in cancer. Adv Cancer Res (2014) 122:281–320. doi: 10.1016/b978-0-12-420117-0.00008-6 24974185

[131] Hasegawa Y Takano T Miyauchi A Matsuzuka F Yoshida H Kuma K. Decreased expression of glutathione peroxidase mRNA in thyroid anaplastic carcinoma. Cancer Lett (2002) 182:69–74. doi: 10.1016/s0304-3835(02)00069-1 12175525

[132] Cazarin J Dupuy C Pires de Carvalho D. Redox homeostasis in thyroid cancer: Implications in Na(+)/I(-) symporter (NIS) regulation. Int J Mol Sci (2022) 23:6129. doi: 10.3390/ijms23116129 35682803 PMC9181215

[133] Cena H Calder PC. Defining a healthy diet: Evidence for the role of contemporary dietary patterns in health and disease. Nutrients (2020) 12:334. doi: 10.3390/nu12020334 32012681 PMC7071223

[134] García-García FJ Monistrol-Mula A Cardellach F Garrabou G. Nutrition, bioenergetics, and metabolic syndrome. Nutrients (2020) 12:2785. doi: 10.3390/nu12092785 32933003 PMC7551996

[135] Rakhra V Galappaththy SL Bulchandani S Cabandugama PK. Obesity and the Western diet: How we got here. Mo Med (2020) 117:536–8. PMC7721435 33311784

[136] Tan BL Norhaizan ME Liew WP. Nutrients and oxidative stress: Friend or foe? Oxid Med Cell Longev (2018) 2018:9719584. doi: 10.1155/2018/9719584 29643982 PMC5831951

[137] Haeiwa H Fujita T Saitoh Y Miwa N. Oleic acid promotes adaptability against oxidative stress in 3T3-L1 cells through lipohormesis. Mol Cell Biochem (2014) 386:73–83. doi: 10.1007/s11010-013-1846-9 24234346

[138] Ravaut G Légiot A Bergeron KF. Monounsaturated fatty acids in obesity-related inflammation. Int J Mol Sci (2020) 22:330. doi: 10.3390/ijms22010330 33396940 PMC7795523

[139] Silva Figueiredo P Carla Inada A Marcelino G Maiara Lopes Cardozo C de Cássia Freitas K de Cássia Avellaneda Guimarães R. Fatty acids consumption: The role metabolic aspects involved in obesity and its associated disorders. Nutrients (2017) 9:1158. doi: 10.3390/nu9101158 29065507 PMC5691774

[140] Meital LT Windsor MT Perissiou M Schulze K Magee R Kuballa A. Omega-3 fatty acids decrease oxidative stress and inflammation in macrophages from patients with small abdominal aortic aneurysm. Sci Rep (2019) 9:12978. doi: 10.1038/s41598-019-49362-z 31506475 PMC6736886

[141] Simopoulos AP. The importance of the omega-6/omega-3 fatty acid ratio in cardiovascular disease and other chronic diseases. Exp Biol Med (Maywood) (2008) 233:674–88. doi: 10.3181/0711-mr-311 18408140

[142] Liu T Qi H Ma L Liu Z Fu H Zhu W. Resveratrol attenuates oxidative stress and extends life span in the annual fish nothobranchius guentheri. Rejuvenation Res (2015) 18:225–33. doi: 10.1089/rej.2014.1618 25569124

[143] Bonnefont-Rousselot D. Resveratrol and cardiovascular diseases. Nutrients (2016) 8:250. doi: 10.3390/nu8050250 27144581 PMC4882663

[144] Dyck GJB Raj P Zieroth S Dyck JRB Ezekowitz JA. The effects of resveratrol in patients with cardiovascular disease and heart failure: A narrative review. Int J Mol Sci (2019) 20:904. doi: 10.3390/ijms20040904 30791450 PMC6413130

[145] Cheng CK Luo JY Lau CW Chen ZY Tian XY Huang Y. Pharmacological basis and new insights of resveratrol action in the cardiovascular system. Br J Pharmacol (2020) 177:1258–77. doi: 10.1111/bph.14801 PMC7056472 31347157

[146] Bo S Ciccone G Castiglione A Gambino R De Michieli F Villois P. Anti-inflammatory and antioxidant effects of resveratrol in healthy smokers a randomized, double-blind, placebo-controlled, cross-over trial. Curr Med Chem (2013) 20:1323–31. doi: 10.2174/0929867311320100009 23298135

[147] Szkudelska K Okulicz M Hertig I Szkudelski T. Resveratrol ameliorates inflammatory and oxidative stress in type 2 diabetic goto-kakizaki rats. BioMed Pharmacother (2020) 125:110026. doi: 10.1016/j.biopha.2020.110026 32092822

[148] Hu HC Lei YH Zhang WH Luo XQ. Antioxidant and anti-inflammatory properties of resveratrol in diabetic nephropathy: A systematic review and meta-analysis of animal studies. Front Pharmacol (2022) 13:841818. doi: 10.3389/fphar.2022.841818 35355720 PMC8959544

[149] Mahjabeen W Khan DA Mirza SA. Role of resveratrol supplementation in regulation of glucose hemostasis, inflammation and oxidative stress in patients with diabetes mellitus type 2: A randomized, placebo-controlled trial. Complement Ther Med (2022) 66:102819. doi: 10.1016/j.ctim.2022.102819 35240291

[150] Su M Zhao W Xu S. Resveratrol in treating diabetes and its cardiovascular complications: A review of its mechanisms of action. Antioxidants (Basel) (2022) 11:1085. doi: 10.3390/antiox11061085 35739982 PMC9219679

[151] Aggarwal BB Bhardwaj A Aggarwal RS Seeram NP Shishodia S Takada Y. Role of resveratrol in prevention and therapy of cancer: preclinical and clinical studies. Anticancer Res (2004) 24:2783–840. 15517885

[152] Rauf A Imran M Butt MS Nadeem M Peters DG Mubarak MS. Resveratrol as an anti-cancer agent: A review. Crit Rev Food Sci Nutr (2018) 58:1428–47. doi: 10.1080/10408398.2016.1263597 28001084

[153] Rotondo S Rajtar G Manarini S Celardo A Rotillo D de Gaetano G. Effect of trans-resveratrol, a natural polyphenolic compound, on human polymorphonuclear leukocyte function. Br J Pharmacol (1998) 123:1691–9. doi: 10.1038/sj.bjp.0701784 PMC1565338 9605577

[154] Mizutani K Ikeda K Kawai Y Yamori Y. Protective effect of resveratrol on oxidative damage in male and female stroke-prone spontaneously hypertensive rats. Clin Exp Pharmacol Physiol (2001) 28:55–9. doi: 10.1046/j.1440-1681.2001.03415.x 11153537

[155] Belguendouz L Frémont L Gozzelino MT. Interaction of transresveratrol with plasma lipoproteins. Biochem Pharmacol (1998) 55:811–6. doi: 10.1016/s0006-2952(97)00544-3 9586953

[156] Wenzel E Soldo T Erbersdobler H Somoza V. Bioactivity and metabolism of trans-resveratrol orally administered to wistar rats. Mol Nutr Food Res (2005) 49:482–94. doi: 10.1002/mnfr.200500003 15779067

[157] Delmas D Jannin B Latruffe N. Resveratrol: preventing properties against vascular alterations and ageing. Mol Nutr Food Res (2005) 49:377–95. doi: 10.1002/mnfr.200400098 15830334

[158] MacCarrone M Lorenzon T Guerrieri P Agrò AF. Resveratrol prevents apoptosis in K562 cells by inhibiting lipoxygenase and cyclooxygenase activity. Eur J Biochem (1999) 265:27–34. doi: 10.1046/j.1432-1327.1999.00630.x 10491155

[159] Leonard SS Xia C Jiang BH Stinefelt B Klandorf H Harris GK. Resveratrol scavenges reactive oxygen species and effects radical-induced cellular responses. Biochem Biophys Res Commun (2003) 309:1017–26. doi: 10.1016/j.bbrc.2003.08.105 13679076

[160] Anand P Kunnumakkara AB Newman RA Aggarwal BB. Bioavailability of curcumin: problems and promises. Mol Pharm (2007) 4:807–18. doi: 10.1021/mp700113r 17999464

[161] Sharifi-Rad J Rayess YE Rizk AA Sadaka C Zgheib R Zam W. Turmeric and its major compound curcumin on health: Bioactive effects and safety profiles for food, pharmaceutical, biotechnological and medicinal applications. Front Pharmacol (2020) 11:1021. doi: 10.3389/fphar.2020.01021 PMC7522354 33041781

[162] Jiang T Ghosh R Charcosset C. Extraction, purification and applications of curcumin from plant materials-a comprehensive review. Trends Food Sci Technol (2021) 112:419–30. doi: 10.1016/j.tifs.2021.04.015

[163] Lin X Bai D Wei Z Zhang Y Huang Y Deng H. Curcumin attenuates oxidative stress in RAW264.7 cells by increasing the activity of antioxidant enzymes and activating the Nrf2-Keap1 pathway. PloS One (2019) 14:e0216711. doi: 10.1371/journal.pone.0216711 31112588 PMC6528975

[164] Menon VP Sudheer AR. Antioxidant and anti-inflammatory properties of curcumin. Adv Exp Med Biol (2007) 595:105–25. doi: 10.1007/978-0-387-46401-5_3 17569207

[165] Maithili Karpaga Selvi N Sridhar MG Swaminathan RP Sripradha R. Curcumin attenuates oxidative stress and activation of redox-sensitive kinases in high fructose- and high-Fat-Fed Male wistar rats. Sci Pharm (2015) 83:159–75. doi: 10.3797/scipharm.1408-16 PMC4727822 26839808

[166] Neag MA Mocan A Echeverria J Pop RM Bocsan CI Crisan G. Berberine: Botanical occurrence, traditional uses, extraction methods, and relevance in cardiovascular, metabolic, hepatic, and renal disorders. Front Pharmacol (2018) 9:557. doi: 10.3389/fphar.2018.00557 30186157 PMC6111450

[167] Li Z Geng YN Jiang JD. Antioxidant and anti-inflammatory activities of berberine in the treatment of diabetes mellitus. Evid Based Complement Alternat Med (2014) 2014:289264. doi: 10.1155/2014/289264 24669227 PMC3942282

[168] Cao RY Zhang Y Feng Z Liu S Liu Y Zheng H. The effective role of natural product berberine in modulating oxidative stress and inflammation related atherosclerosis: Novel insights into the gut-heart axis evidenced by genetic sequencing analysis. Front Pharmacol (2021) 12:764994. doi: 10.3389/fphar.2021.764994 35002703 PMC8727899

[169] Ma X Chen Z Wang L Wang G Wang Z Dong X. The pathogenesis of diabetes mellitus by oxidative stress and inflammation: Its inhibition by berberine. Front Pharmacol (2018) 9:782. doi: 10.3389/fphar.2018.00782 30100874 PMC6072898

[170] Pizzino G Irrera N Cucinotta M Pallio G Mannino F Arcoraci V. Oxidative stress: Harms and benefits for human health. Oxid Med Cell Longev (2017) 2017:8416763. doi: 10.1155/2017/8416763 28819546 PMC5551541

[171] Aceves C Anguiano B Delgado G. The extrathyronine actions of iodine as antioxidant, apoptotic, and differentiation factor in various tissues. Thyroid (2013) 23:938–46. doi: 10.1089/thy.2012.0579 PMC3752513 23607319

[172] Zicker S Schoenherr B. Focus on nutrition: the role of iodine in nutrition and metabolism. Compend Contin Educ Vet (2012) 34:E1–4. 23532759

[173] Luo J Hendryx M Dinh P He K. Association of iodine and iron with thyroid function. Biol Trace Elem Res (2017) 179:38–44. doi: 10.1007/s12011-017-0954-x 28160243

[174] Zimmermann MB Galetti V. Iodine intake as a risk factor for thyroid cancer: a comprehensive review of animal and human studies. Thyroid Res (2015) 8:8. doi: 10.1186/s13044-015-0020-8 26146517 PMC4490680

[175] Wolff J Chaikoff IL. Plasma inorganic iodide as a homeostatic regulator of thyroid function. J Biol Chem (1948) 174:555–64. doi: 10.1016/S0021-9258(18)57335-X 18865621

[176] Leung AM Braverman LE. Consequences of excess iodine. Nat Rev Endocrinol (2014) 10:136–42. doi: 10.1038/nrendo.2013.251 PMC3976240 24342882

[177] Civitareale D Saiardi A Falasca P. Purification and characterization of thyroid transcription factor 2. Biochem J (1994) 304(Pt 3):981–5. doi: 10.1042/bj3040981 PMC1137428 7818505

[178] Severo JS Morais JBS de Freitas TEC Andrade ALP Feitosa MM Fontenelle LC. The role of zinc in thyroid hormones metabolism. Int J Vitam Nutr Res (2019) 89:80–8. doi: 10.1024/0300-9831/a000262 30982439

[179] Kralik A Eder K Kirchgessner M. Influence of zinc and selenium deficiency on parameters relating to thyroid hormone metabolism. Horm Metab Res (1996) 28:223–6. doi: 10.1055/s-2007-979169 8738110

[180] Nishi Y Kawate R Usui T. Zinc metabolism in thyroid disease. Postgrad Med J (1980) 56:833–7. doi: 10.1136/pgmj.56.662.833 PMC2424839 7267493

[181] Drutel A Archambeaud F Caron P. Selenium and the thyroid gland: more good news for clinicians. Clin Endocrinol (Oxf) (2013) 78:155–64. doi: 10.1111/cen.12066 23046013

[182] Köhrle J. Selenium and the thyroid. Curr Opin Endocrinol Diabetes Obes (2013) 20:441–8. doi: 10.1097/01.med.0000433066.24541.88 23974773

[183] Zoidis E Seremelis I Kontopoulos N Danezis GP. Selenium-dependent antioxidant enzymes: Actions and properties of selenoproteins. Antioxidants (Basel) (2018) 7:66. doi: 10.3390/antiox7050066 29758013 PMC5981252

[184] Giuliani C Iezzi M Ciolli L Hysi A Bucci I Di Santo S. Resveratrol has anti-thyroid effects both in vitro and in vivo. Food Chem Toxicol (2017) 107:237–47. doi: 10.1016/j.fct.2017.06.044 28668442

[185] Han Z Cen C Ou Q Pan Y Zhang J Huo D. The potential prebiotic berberine combined with methimazole improved the therapeutic effect of graves’ disease patients through regulating the intestinal microbiome. Front Immunol (2021) 12:826067. doi: 10.3389/fimmu.2021.826067 35082799 PMC8785824

[186] Benvenga S Lakshmanan M Trimarchi F. Carnitine is a naturally occurring inhibitor of thyroid hormone nuclear uptake. Thyroid (2000) 10:1043–50. doi: 10.1089/thy.2000.10.1043 11201848

[187] Benvenga S Nordio M Laganà AS Unfer V. The role of inositol in thyroid physiology and in subclinical hypothyroidism management. Front Endocrinol (Lausanne) (2021) 12:662582. doi: 10.3389/fendo.2021.662582 34040582 PMC8143049

[188] Spaggiari G Brigante G De Vincentis S Cattini U Roli L De Santis MC. Probiotics ingestion does not directly affect thyroid hormonal parameters in hypothyroid patients on levothyroxine treatment. Front Endocrinol (Lausanne) (2017) 8:316. doi: 10.3389/fendo.2017.00316 29184537 PMC5694461

[189] Fröhlich E Wahl R. Microbiota and thyroid interaction in health and disease. Trends Endocrinol Metab (2019) 30:479–90. doi: 10.1016/j.tem.2019.05.008 31257166

[190] Kiełczykowska M Kocot J Paździor M Musik I. Selenium - a fascinating antioxidant of protective properties. Adv Clin Exp Med (2018) 27:245–55. doi: 10.17219/acem/67222 29521069

[191] Hu S Rayman MP. Multiple nutritional factors and the risk of hashimoto’s thyroiditis. Thyroid (2017) 27:597–610. doi: 10.1089/thy.2016.0635 28290237

[192] Rasmussen LB Schomburg L Köhrle J Pedersen IB Hollenbach B Hög A. Selenium status, thyroid volume, and multiple nodule formation in an area with mild iodine deficiency. Eur J Endocrinol (2011) 164:585–90. doi: 10.1530/eje-10-1026 21242171

[193] Huang Z Rose AH Hoffmann PR. The role of selenium in inflammation and immunity: from molecular mechanisms to therapeutic opportunities. Antioxid Redox Signal (2012) 16:705–43. doi: 10.1089/ars.2011.4145 PMC3277928 21955027

[194] Toulis KA Anastasilakis AD Tzellos TG Goulis DG Kouvelas D. Selenium supplementation in the treatment of hashimoto’s thyroiditis: a systematic review and a meta-analysis. Thyroid (2010) 20:1163–73. doi: 10.1089/thy.2009.0351 20883174

[195] Pizzulli A Ranjbar A. Selenium deficiency and hypothyroidism: a new etiology in the differential diagnosis of hypothyroidism in children. Biol Trace Elem Res (2000) 77:199–208. doi: 10.1385/bter:77:3:199 11204462

[196] Burk RF Hill KE. Regulation of selenium metabolism and transport. Annu Rev Nutr (2015) 35:109–34. doi: 10.1146/annurev-nutr-071714-034250 25974694

[197] Sebai H Hovsépian S Ristorcelli E Aouani E Lombardo D Fayet G. Resveratrol increases iodide trapping in the rat thyroid cell line FRTL-5. Thyroid (2010) 20:195–203. doi: 10.1089/thy.2009.0171 20151827

[198] Shih A Davis FB Lin HY Davis PJ. Resveratrol induces apoptosis in thyroid cancer cell lines *via* a MAPK- and p53-dependent mechanism. J Clin Endocrinol Metab (2002) 87:1223–32. doi: 10.1210/jcem.87.3.8345 11889192

[199] Truong M Cook MR Pinchot SN Kunnimalaiyaan M Chen H. Resveratrol induces Notch2-mediated apoptosis and suppression of neuroendocrine markers in medullary thyroid cancer. Ann Surg Oncol (2011) 18:1506–11. doi: 10.1245/s10434-010-1488-z PMC3078954 21184191

[200] Benvenga S Antonelli A. Inositol(s) in thyroid function, growth and autoimmunity. Rev Endocr Metab Disord (2016) 17:471–84. doi: 10.1007/s11154-016-9370-3 27315814

[201] Nordio M Pajalich R. Combined treatment with myo-inositol and selenium ensures euthyroidism in subclinical hypothyroidism patients with autoimmune thyroiditis. J Thyroid Res (2013) 2013:424163. doi: 10.1155/2013/424163 24224112 PMC3809375

[202] Paparo SR Ferrari SM Patrizio A Elia G Ragusa F Botrini C. Myoinositol in autoimmune thyroiditis. Front Endocrinol (Lausanne) (2022) 13:930756. doi: 10.3389/fendo.2022.930756 35837308 PMC9273877

[203] Nordio M Basciani S. Treatment with myo-inositol and selenium ensures euthyroidism in patients with autoimmune thyroiditis. Int J Endocrinol (2017) 2017:2549491. doi: 10.1155/2017/2549491 28293260 PMC5331475

[204] Nordio M Basciani S. Myo-inositol plus selenium supplementation restores euthyroid state in hashimoto’s patients with subclinical hypothyroidism. Eur Rev Med Pharmacol Sci (2017) 21:51–9. 28724185

[205] Nordio M Basciani S. Evaluation of thyroid nodule characteristics in subclinical hypothyroid patients under a myo-inositol plus selenium treatment. Eur Rev Med Pharmacol Sci (2018) 22:2153–9. doi: 10.26355/eurrev_201804_14749 29687875

[206] Pekala J Patkowska-Sokoła B Bodkowski R Jamroz D Nowakowski P Lochyński S. L-carnitine–metabolic functions and meaning in humans life. Curr Drug Metab (2011) 12:667–78. doi: 10.2174/138920011796504536 21561431

[207] Gülçin I. Antioxidant and antiradical activities of l-carnitine. Life Sci (2006) 78:803–11. doi: 10.1016/j.lfs.2005.05.103 16253281

[208] Wang ZY Liu YY Liu GH Lu HB Mao CY. L-carnitine and heart disease. Life Sci (2018) 194:88–97. doi: 10.1016/j.lfs.2017.12.015 29241711

[209] Gnoni A Longo S Gnoni GV Giudetti AM. Carnitine in human muscle bioenergetics: Can carnitine supplementation improve physical exercise? Molecules (2020) 25:182. doi: 10.3390/molecules25010182 31906370 PMC6982879

[210] Benvenga S Ruggeri RM Russo A Lapa D Campenni A Trimarchi F. Usefulness of l-carnitine, a naturally occurring peripheral antagonist of thyroid hormone action, in iatrogenic hyperthyroidism: a randomized, double-blind, placebo-controlled clinical trial. J Clin Endocrinol Metab (2001) 86:3579–94. doi: 10.1210/jcem.86.8.7747 11502782

[211] Benvenga S Lapa D Cannavò S Trimarchi F. Successive thyroid storms treated with l-carnitine and low doses of methimazole. Am J Med (2003) 115:417–8. doi: 10.1016/s0002-9343(03)00399-1 14553887

[212] Kimmoun A Munagamage G Dessalles N Gerard A Feillet F Levy B. Unexpected awakening from comatose thyroid storm after a single intravenous injection of l-carnitine. Intensive Care Med (2011) 37:1716–7. doi: 10.1007/s00134-011-2293-2 21739342

[213] Chee R Agah R Vita R Benvenga S. L-carnitine treatment in a seriously ill cancer patient with severe hyperthyroidism. Hormones (Athens) (2014) 13:407–12. doi: 10.14310/horm.2002.1494 25079466

[214] Maebashi M Kawamura N Sato M Imamura A Yoshinaga K. Urinary excretion of carnitine in patients with hyperthyroidism and hypothyroidism: augmentation by thyroid hormone. Metabolism (1977) 26:351–6. doi: 10.1016/0026-0495(77)90101-9 846404

[215] Sinclair C Gilchrist JM Hennessey JV Kandula M. Muscle carnitine in hypo- and hyperthyroidism. Muscle Nerve (2005) 32:357–9. doi: 10.1002/mus.20336 15803480

[216] An JH Kim YJ Kim KJ Kim SH Kim NH Kim HY. L-carnitine supplementation for the management of fatigue in patients with hypothyroidism on levothyroxine treatment: a randomized, double-blind, placebo-controlled trial. Endocr J (2016) 63:885–95. doi: 10.1507/endocrj.EJ16-0109 27432821

[217] Hill C Guarner F Reid G Gibson GR Merenstein DJ Pot B. Expert consensus document. the international scientific association for probiotics and prebiotics consensus statement on the scope and appropriate use of the term probiotic. Nat Rev Gastroenterol Hepatol (2014) 11:506–14. doi: 10.1038/nrgastro.2014.66 24912386

[218] Mishra V Shah C Mokashe N Chavan R Yadav H Prajapati J. Probiotics as potential antioxidants: a systematic review. J Agric Food Chem (2015) 63:3615–26. doi: 10.1021/jf506326t 25808285

[219] Gomes AC Bueno AA de Souza RG Mota JF. Gut microbiota, probiotics and diabetes. Nutr J (2014) 13:60. doi: 10.1186/1475-2891-13-60 24939063 PMC4078018

[220] Wang J Tang H Zhang C Zhao Y Derrien M Rocher E. Modulation of gut microbiota during probiotic-mediated attenuation of metabolic syndrome in high fat diet-fed mice. Isme J (2015) 9:1–15. doi: 10.1038/ismej.2014.99 24936764 PMC4274436

[221] Rad AH Abbasalizadeh S Vazifekhah S Abbasalizadeh F Hassanalilou T Bastani P. The future of diabetes management by healthy probiotic microorganisms. Curr Diabetes Rev (2017) 13:582–9. doi: 10.2174/1573399812666161014112515 27758705

[222] Asemi Z Zare Z Shakeri H Sabihi SS Esmaillzadeh A. Effect of multispecies probiotic supplements on metabolic profiles, hs-CRP, and oxidative stress in patients with type 2 diabetes. Ann Nutr Metab (2013) 63:1–9. doi: 10.1159/000349922 23899653

[223] Wang Y Wu Y Wang Y Xu H Mei X Yu D. Antioxidant properties of probiotic bacteria. Nutrients (2017) 9:521. doi: 10.3390/nu9050521 28534820 PMC5452251

[224] Wang AN Yi XW Yu HF Dong B Qiao SY. Free radical scavenging activity of lactobacillus fermentum *in vitro* and its antioxidative effect on growing-finishing pigs. J Appl Microbiol (2009) 107:1140–8. doi: 10.1111/j.1365-2672.2009.04294.x 19486423

[225] Stecchini ML Del Torre M Munari M. Determination of peroxy radical-scavenging of lactic acid bacteria. Int J Food Microbiol (2001) 64:183–8. doi: 10.1016/s0168-1605(00)00456-6 11252501

[226] Kullisaar T Zilmer M Mikelsaar M Vihalemm T Annuk H Kairane C. Two antioxidative lactobacilli strains as promising probiotics. Int J Food Microbiol (2002) 72:215–24. doi: 10.1016/s0168-1605(01)00674-2 11845820

[227] Ejtahed HS Mohtadi-Nia J Homayouni-Rad A Niafar M Asghari-Jafarabadi M Mofid V. Probiotic yogurt improves antioxidant status in type 2 diabetic patients. Nutrition (2012) 28:539–43. doi: 10.1016/j.nut.2011.08.013 22129852

[228] Martarelli D Verdenelli MC Scuri S Cocchioni M Silvi S Cecchini C. Effect of a probiotic intake on oxidant and antioxidant parameters in plasma of athletes during intense exercise training. Curr Microbiol (2011) 62:1689–96. doi: 10.1007/s00284-011-9915-3 21400082

[229] Zilmer M Soomets U Rehema A Langel U. The glutathione system as an attractive therapeutic target. Drug Design Reviews-Online (Discontinued) (2005) 2:121–7. doi: 10.2174/1567269053202697

[230] Bäckhed F Ley RE Sonnenburg JL Peterson DA Gordon JI. Host-bacterial mutualism in the human intestine. Science (2005) 307:1915–20. doi: 10.1126/science.1104816 15790844

[231] Berg RD. The indigenous gastrointestinal microflora. Trends Microbiol (1996) 4:430–5. doi: 10.1016/0966-842x(96)10057-3 8950812

[232] Rajilić-Stojanović M Smidt H de Vos WM. Diversity of the human gastrointestinal tract microbiota revisited. Environ Microbiol (2007) 9:2125–36. doi: 10.1111/j.1462-2920.2007.01369.x 17686012

[233] Hsiao WW Metz C Singh DP Roth J. The microbes of the intestine: an introduction to their metabolic and signaling capabilities. Endocrinol Metab Clin North Am (2008) 37:857–71. doi: 10.1016/j.ecl.2008.08.006 PMC4411945 19026936

[234] Frank DN St Amand AL Feldman RA Boedeker EC Harpaz N Pace NR. Molecular-phylogenetic characterization of microbial community imbalances in human inflammatory bowel diseases. Proc Natl Acad Sci U.S.A. (2007) 104:13780–5. doi: 10.1073/pnas.0706625104 PMC1959459 17699621

[235] Zhang J Zhang F Zhao C Xu Q Liang C Yang Y. Dysbiosis of the gut microbiome is associated with thyroid cancer and thyroid nodules and correlated with clinical index of thyroid function. Endocrine (2019) 64:564–74. doi: 10.1007/s12020-018-1831-x 30584647

[236] Zitvogel L Ayyoub M Routy B Kroemer G. Microbiome and anticancer immunosurveillance. Cell (2016) 165:276–87. doi: 10.1016/j.cell.2016.03.001 27058662

[237] Rajagopala SV Vashee S Oldfield LM Suzuki Y Venter JC Telenti A. The human microbiome and cancer. Cancer Prev Res (Phila) (2017) 10:226–34. doi: 10.1158/1940-6207.capr-16-0249 28096237

[238] Benitez AJ Hoffmann C Muir AB Dods KK Spergel JM Bushman FD. Inflammation-associated microbiota in pediatric eosinophilic esophagitis. Microbiome (2015) 3:23. doi: 10.1186/s40168-015-0085-6 26034601 PMC4450515

[239] Dahmus JD Kotler DL Kastenberg DM Kistler CA. The gut microbiome and colorectal cancer: a review of bacterial pathogenesis. J Gastrointest Oncol (2018) 9:769–77. doi: 10.21037/jgo.2018.04.07 PMC6087872 30151274

[240] Trapani KM Boghossian LJ. Clostridium subterminale septicemia in a patient with metastatic gastrointestinal adenocarcinoma. Case Rep Infect Dis (2018) 2018:6031510. doi: 10.1155/2018/6031510 29951328 PMC5987309

[241] Virili C Fallahi P Antonelli A Benvenga S Centanni M. Gut microbiota and hashimoto’s thyroiditis. Rev Endocr Metab Disord (2018) 19:293–300. doi: 10.1007/s11154-018-9467-y 30294759

[242] Wu Q Rayman MP Lv H Schomburg L Cui B Gao C. Low population selenium status is associated with increased prevalence of thyroid disease. J Clin Endocrinol Metab (2015) 100:4037–47. doi: 10.1210/jc.2015-2222 26305620

[243] Bülow Pedersen I Knudsen N Carlé A Schomburg L Köhrle J Jørgensen T. Serum selenium is low in newly diagnosed graves’ disease: a population-based study. Clin Endocrinol (Oxf) (2013) 79:584–90. doi: 10.1111/cen.12185 23448365

[244] Schomburg L. Selenium deficiency due to diet, pregnancy, severe illness, or COVID-19-A preventable trigger for autoimmune disease. Int J Mol Sci (2021) 22:8532. doi: 10.3390/ijms22168532 34445238 PMC8395178

[245] Krassas GE Pontikides N Tziomalos K Tzotzas T Zosin I Vlad M. Selenium status in patients with autoimmune and non-autoimmune thyroid diseases from four European countries. Expert Rev Endocrinol Metab (2014) 9:685–92. doi: 10.1586/17446651.2014.960845 30736204

[246] Gorini F Sabatino L. Selenium: An element of life essential for thyroid function. Molecules (2021) 26:7084. doi: 10.3390/molecules26237084 34885664 PMC8658851

